# Prevalence, Antimicrobial Resistance Profiles, and Molecular Characteristics of Methicillin-Resistant *Staphylococcus aureus* Among School Children in Nha Trang, Central Vietnam

**DOI:** 10.3390/pathogens15020238

**Published:** 2026-02-22

**Authors:** Stephen Anyona Omae, Shah Mohammad Monir, Hien-Anh Thi Nguyen, Kim-Mai Huynh, Natsuki Ariyoshi, Lien Thuy Le, Dat Thanh Le, Trieu Bao Nguyen, Hoang Huy Le, Luong Dinh Nguyen, Miyuki Tsuruoka, Hirono Otomaru, Erik Koehne, Michiko Toizumi, Duc-Anh Dang, Hung Thai Do, Lay-Myint Yoshida

**Affiliations:** 1 Department of Pediatric Infectious Diseases, Institute of Tropical Medicine, Nagasaki University, Nagasaki 852-8523, Japan; sanyona8@gmail.com (S.A.O.); shah@nagasaki-u.ac.jp (S.M.M.); kinoko.ariyoshi@gmail.com (N.A.); dr.dinhluong86@gmail.com (L.D.N.); m-tsuruoka@nagasaki-u.ac.jp (M.T.); otomaru-h@nagasaki-u.ac.jp (H.O.); erik.koehne@nagasaki-u.ac.jp (E.K.); toizumi@nagasaki-u.ac.jp (M.T.); hungdt02@yahoo.com (H.T.D.); 2 Graduate School of Biomedical Sciences, Nagasaki University, Nagasaki 852-8523, Japan; 3 Center for Microbiology Research, Kenya Medical Research Institute, Nairobi 54840-00200, Kenya; 4 National Institute of Hygiene and Epidemiology, Hanoi 100000, Vietnam; hienanh75@yahoo.com (H.-A.T.N.); lehuyhoang2010@gmail.com (H.H.L.); dangducanh.nihe@gmail.com (D.-A.D.); 5 Pasteur Institute in Nha Trang, 06-08-10 Tran Phu Street, Van Thanh Ward, Nha Trang 650000, Vietnam; kimanhlt88@gmail.com (K.-M.H.); lethuylien250786@gmail.com (L.T.L.); lethanhdat89dk@yahoo.com.vn (D.T.L.); baotrieuipn@yahoo.com.vn (T.B.N.); 6 School for Tropical Medicine and Global Health, Nagasaki University, Nagasaki 852-8523, Japan

**Keywords:** *Staphylococcus aureus*, methicillin-resistant *Staphylococcus aureus*, antibiotic resistance, multi-locus sequence typing, Vietnam

## Abstract

*Staphylococcus aureus* (*S. aureus*), especially Methicillin-resistant *Staphylococcus aureus* (MRSA), remains a major public health concern both in hospital and community settings. The MRSA carriage situation among schoolchildren in Vietnam is limited. A cross-sectional study was conducted in March 2023 to assess the prevalence, antimicrobial resistance patterns, and molecular characteristics of MRSA among schoolchildren in Nha Trang, Vietnam. Schoolchildren from grades 1 to 12 (ages 6–18 yrs) were enrolled. Background epidemiological data and nasal swabs were collected. Nasal samples were processed at the Pasteur Institute in Nha Trang, Vietnam, for *S. aureus* culture. Out of 1210 participants enrolled, *S. aureus* prevalence was 18.3% (222/1210), of which 41% (91/222) were MRSA. Primary school children showed the highest prevalence of MRSA colonization (48%), 32.8% in secondary, and 27.8% in high school. Among MRSA isolates, high levels of resistance were detected against trimethoprim-sulfamethoxazole (100%), erythromycin (68.2%) and clindamycin (45.1%). Multidrug resistance (MDR) occurred in 30% (27/90) of MRSA isolates. *Staphylococcal* cassette chromosome *mec* (SCC*mec)* subtype IVa was dominant (66.0%), followed by type IV (7.0%) and type V (6.0%). MLST data revealed genetic diversity whereby ST45 dominated, followed by ST546 and ST188. Continuous MRSA surveillance is essential to monitor emerging strains in the communities.

## 1. Introduction

*Staphylococcus aureus* (*S. aureus*) is a Gram-positive coccus that appears in grape-like clusters under microscopy [1]. Although it is a common commensal organism in humans, *S. aureus* is an important pathogen capable of causing a wide spectrum of diseases ranging from mild skin and soft tissue infections to severe and potentially fatal conditions, such as pneumonia, endocarditis, osteomyelitis, and sepsis [2]. *S*. *aureus* contributes substantially to mortality in resource-constrained regions of the Asia Pacific, including Vietnam; however, data on its colonization and associated disease burden remain scarce [3].

Nasal colonization plays a central role in the epidemiology of *S. aureus*. Approximately 30% of healthy individuals are persistent or intermittent nasal carriers of *S. aureus*, with the nose serving as the primary ecological niche for bacterial colonization [4]. School-aged children represent a particularly important population for *S. aureus* transmission. High colonization rates have been reported among children worldwide, with prevalence varying substantially across geographic regions and populations. Studies from Nepal and Vietnam have reported *S. aureus* carriage rates of approximately 15% and 29.8%, respectively, in the upper respiratory tract of children [5]. Close contact environments, such as schools, facilitate bacterial spread through frequent physical contact, sharing of personal items, and suboptimal hygiene practices [6]. School overcrowding comprises hygiene infrastructure and increases close contact, creating conditions that favor sustained MRSA transmission among school children. Consequently, schoolchildren may act as reservoirs for community dissemination of both susceptible and antibiotic-resistant strains.

The emergence and global spread of antibiotic-resistant *S. aureus*, particularly Methicillin-resistant *S. aureus* (MRSA), has become a major public health concern. Antibiotic resistance arises through the acquisition of resistance genes, mutations in housekeeping genes, and selective pressure exerted by antibiotic misuse and overuse [7]. Early resistance in *S. aureus* was driven by the production of penicillinase, which hydrolyzes the β-lactam ring of penicillin. Methicillin, a semisynthetic β-lactam resistant to β-lactamase, was subsequently developed; however, MRSA strains emerged shortly after its introduction [8].

Methicillin resistance is mediated primarily by the acquisition of the *mecA* gene, which encodes an altered penicillin-binding protein (PBP2a) with low affinity for β-lactam antibiotics, enabling bacterial survival despite antibiotic exposure [9]. Since the 1980s, MRSA has spread globally, causing both hospital- and community-associated infections, with some countries reporting methicillin resistance in more than 50% of *S. aureus* isolates [10].

In Vietnam, MRSA accounts for a substantial proportion of *S. aureus* infections, with estimates indicating approximately 74% of hospital-acquired and 30% of community-acquired cases [11]. MRSA strains are broadly classified into hospital-associated (HA-MRSA) and community-associated (CA-MRSA). HA-MRSA strains are typically multidrug resistant (MDR), defined as resistance to at least one agent in three or more antimicrobial classes, limiting therapeutic options and increasing morbidity and mortality [12]. In contrast, CA-MRSA strains often exhibit enhanced virulence and may harbor toxins such as Panton–Valentine leukocidin (PVL), which is associated with severe skin, soft tissue, and necrotizing pulmonary infections.

Molecular characterization is essential for understanding MRSA epidemiology. MRSA strains are defined by the type of staphylococcal cassette chromosome *mec* (SCCmec) [13]. Molecular typing methods such as SCCmec typing, multi-locus sequence typing (MLST), and virulence gene profiling, including detection of *lukS-PV* and *lukF-PV*, provide valuable insights into strain diversity, transmission dynamics, and pathogenic potential [14].

Despite the public health importance of MRSA, data on nasal carriage, antimicrobial resistance profiles, and molecular characteristics among Vietnamese schoolchildren remain scarce. Monitoring *S. aureus* and MRSA colonization in this population is critical, as colonized children may serve as reservoirs for respiratory and invasive infections and facilitate community-wide transmission. Given the rising global threat of antimicrobial resistance and its associated clinical and economic burden, targeted surveillance in school settings is essential. Therefore, this study aimed to assess the prevalence, antimicrobial resistance patterns, and molecular characteristics of MRSA among schoolchildren in Nha Trang, Central Vietnam.

## 2. Materials and Methods

### 2.1. Study Design and Setting

A cross-sectional study was conducted in March 2023, in Nha Trang, Vietnam. Nha Trang, the capital of Khanh Hoa Province, is on the south-central coast of Vietnam. This site is an ideal location for community-based surveillance studies because the city’s eastern side borders the sea, and the other sides are surrounded by mountains.

### 2.2. Study Population

Schoolchildren aged 6 to 18 years (grades 1–12) who were asymptomatic and apparently healthy and residing in Nha Trang, Vietnam, were enrolled in the study, with recruitment conducted primarily through commune health centers in four selected communes (two urban and two rural). Based on previous census data, the approximate numbers of primary, secondary, and high school-aged children in Nha Trang city were 15,000, 12,000, and 9000 respectively. As an exploratory study, we aimed to enroll 100 schoolchildren per grade.

### 2.3. Inclusion Criteria

- The student was healthy and did not have any chronic illnesses such as asthma, congenital heart disease, etc.
- The student who did not have any febrile illness within the last month.
- For primary school students, participation required informed consent from a parent or guardian. For secondary and high school students, participation required the student’s own agreement to provide a sample and take part in the study.

The study did not include participants who did not meet at least one of the inclusion criteria listed above.

### 2.4. Data Collection

After obtaining consent during a home visit, the schoolchildren were invited to the nearby local community health center on a weekend, outside of school hours, for enrollment. Briefly, the study physician explained the aim of the study to the parents/guardians and the students. The study physician conducted clinical examinations on the participants to ensure they were in good health and eligible to participate in the study. Informed written consent was obtained from parents/legal guardians and assent from older children before data collection. A pretested questionnaire written in English and translated into Vietnamese was used to collect background epidemiological information. A study identification number was assigned to each subject, and all the information obtained from the study participants was coded to maintain confidentiality.

### 2.5. Sample Collection

Nasal swabs were collected by the study physician using a flexible flocked swab. Gently, a swab was inserted two to three centimeters into the front of each nostril of both anterior nares of each participant and turned four to five times in both clockwise and counterclockwise directions. The nasal swabs were placed into screw-capped test tubes containing Skim Milk–Tryptone–Glucose–Glycerol (STGG) medium. The swabs were labeled, packaged in insulated containers with ice packs, and transported within the recommended time frame to the microbiology laboratory at Pasteur Institute in Nha Trang for processing and storage according to standard microbiological procedures. A portion of the nasal samples was put in STGG medium and shipped to the Pediatric Infectious Diseases Department (PID), Institute of Tropical Medicine (Nekken), Nagasaki University, Japan, for further confirmatory testing and genotypic processes.

### 2.6. Bacterial Isolation and Identification

The nasal samples were inoculated on 5% sheep blood agar (SBA) plates and incubated at 35–37 °C for 24 h. Based on colony morphology, β-hemolytic colonies on SBA were subcultured onto mannitol salt agar (MSA) media (HiMedia, Mumbai, India). Yellow colonies on MSA were further examined by Gram stain, catalase, and coagulase tests for confirmation. Gram-positive cocci, arranged in grape-like clusters, catalase, and coagulase-positive were recorded as *S. aureus*. All isolates were further confirmed by the detection of the *nuc*A gene by PCR for *S. aureus* and the detection of *mec*A for MRSA [15]. Next, these isolates were characterized by minimum inhibitory concentration (MIC) and sequence type (ST) at the Pediatric Infectious Diseases Department laboratory, Nekken. All procedures were carried out following standard microbiological procedures as previously described [16].

### 2.7. Antibiotic Susceptibility Testing

The phenotypic characterization to assess the antimicrobial resistance pattern of all 222 *S. aureus* isolates was subjected to an antibiotic susceptibility test against commonly used antibiotics, as well as standard treatment guidelines for the treatment of *S. aureus* infections. Antibiotic panel used included Ampicillin (AMP), Daptomycin (DAP), Imipenem (IPM), Nitrofurantoin (NI/F), Gentamicin (GEN), Erythromycin (ERY), Tetracycline (TCY), Tigecycline (TGC), Ciprofloxacin (CIP), Levofloxacin (LVX), Moxifloxacin (MFX), Clindamycin (CLI), Rifampin (RIF), Trimethoprim-Sulfamethoxazole (SXT), Quinupristin/Dalfopristin (Q/D), Linezolid (LZD), and Vancomycin (VAN) using the VITEK2 system (bioMérieux, France). MRSA screening was performed using cefoxitin (30 μg) strips via the strip diffusion method [17]. Isolates showing zones of inhibition of 22 mm or greater were classified as phenotypically Methicillin-sensitive *S. aureus* (MSSA), while those with zones <22 mm were classified as Methicillin-resistant *S. aureus* (MRSA), with confirmation based on the presence of the *mecA* gene. The Minimum Inhibitory Concentration (MIC) for Ampicillin, Daptomycin, and Imipenem was determined by the E-test method. Multidrug resistance (MDR) was defined as resistance to three or more distinct classes of antimicrobial drugs, and the control strain used was *S. aureus* ATCC 25923 [12]. The procedure and interpretation of results were carried out according to Clinical and Laboratory Standards Institute (CLSI) M100-S31, 2023, and EUCAST 2023 guidelines.

### 2.8. Molecular Analysis

#### 2.8.1. Genomic DNA Extraction

Genomic DNA extraction was done using DNeasy Blood and Tissue Kit (Qiagen GmbH, Hilden, Germany) (250) according to the manufacturer’s instructions. DNA concentration and quality assessment were determined using a NanoDrop spectrometer (Thermo Scientific, Waltham, MA, USA). The extracted DNA was stored at −30 °C for later analysis.

#### 2.8.2. Genotypical Identification

To confirm the identity of the isolates at the genetic level, *nucA* PCR was performed to detect the thermonuclease gene, which is a species-specific gene to *S. aureus,* using primers shown to amplify a 165 bp fragment. NucA-F (5′-CGGGTCCTTTCAAAAAGGGGA-3′) and NucA-R (5′-TCACCGTTTCTGGCGTATCA-3′) [18]. A commercial *S. aureus* ATCC 25923 was used as a positive control. MRSA screening was done by PCR to detect the *mec*A gene (297 bp) using *mec*A-F (5′-TGGCTCAGGTACTGCTATCCA-3′) and *mec*A-R (5′-ACGTTGTAACCACCCCAAGAT-3′) primers. Multiplex PCR reactions were performed in a final volume of 25 µL containing 1× PCR buffer, 2 mM MgCl_2_, 0.2 mM dNTPs, 10 pmol of each primer, and 50 ng of genomic DNA. ATCC 43300 was used as a positive control. The thermal cycling conditions included an initial denaturation at 94 °C for 5 min; followed by 30 cycles of denaturation at 94 °C for 45 s, annealing at 58 °C for 45 s, followed by extension at 72 °C for 45 s, and a final extension at 72 °C for 5 min [17].

#### 2.8.3. Determination of PVL Gene

Multiplex PCR assay was used for the detection of the PVL gene using the following set of primers: Luk-PV-1 (5′-ATCATTAGGTAAAATGTCTGGACATGATCCAGC-′3) and Luk-PV-2 (5′-GCATCAAGTGTATTGGATAGCAAAAGCGC-′3) [19]. The PCR mixture had a total volume of 25 µL, consisting of 2 µL of DNA template, AmpliTaq PCR buffer, 1.5 mM MgCl_2_, 100 µM of each dNTP, 0.4 µM of each primer (pvl-F and pvl-R), together with 1.25 U of AmpliTaq DNA polymerase (Thermo Fisher Scientific, Waltham, MA, USA). Thermal cycling conditions were as follows: denaturation at 94 °C for 5 min; followed by 28 cycles of 94 °C for 1 min, and 55 °C for 1 min, and 1 min, and 72 °C for 1 min; with a final extension at 72 °C for 10 min. A laboratory strain previously known to be positive for the *PVL* gene was used as a positive control, and a negative control strain was also used with each run, as previously described [20].

#### 2.8.4. Determination of *SCCmec* Gene Types

SCC*mec* gene identification I–V was carried out as described previously by Zhang et al. [21]. The primers used for SCC*mec* are listed in the Supplementary Material.

#### 2.8.5. Multi-Locus Sequence Typing (MLST)

MLST analysis was performed on all 222 *S. aureus* isolates through PCR amplification of seven housekeeping genes: carbamate (*arc*C), shikimate dehydrogenase (*aroE*), glycerol kinase (*glpF*), guanylate kinase (*gmk*), phosphate acetyltransferase (*pta*), triosephosphate isomerase (*tpi*), and acetyl coenzyme A acetyltransferase (*yqiL*) as previously described [22]. PCR reactions were carried out using AmpliTaq Gold Master mixes (Thermo Fisher Scientific, Waltham, MA, USA).

### 2.9. Ethical Approval

This study was approved by the National Institute of Hygiene and Epidemiology (NIHE) Ethical Review Committee, Hanoi, Vietnam (VN01057/IORG), and Nagasaki University, Japan (approval number: 221215285), respectively. To minimize bias and ensure transparency, the study objectives were clearly explained and written informed consent was obtained from all participants’ parents/guardians prior to enrollment and sample collection.

### 2.10. Data Analysis

The statistical software, R (R-studio, version 2025.09.2+418), was used for data analysis. VectorBee software (version 2.7.0) was used to clean and assemble the sequences into consensus files, and MEGA 12 software was used to construct the maximum likelihood phylogenetic tree. Alleles and STs were analyzed and determined by the official *S. aureus* MLST database (http://saureus.mlst.net) (last accessed on 15 December 2025) and bioinformatic tools (blastn and fastmlst). Phylogenetic tree visualization was generated in R using the ggplot2 package. Bootstrap analysis was performed using 1000 replicates, and values ≥ 70% were considered significant.

Descriptive statistics, including means, standard deviations, frequencies, and percentages, were calculated for the sociodemographic variables. Univariate analysis was performed to identify risk factors associated with *S. aureus* colonization, using Pearson’s (χ^2^) test or Fisher’s exact test, as appropriate. Univariate analyses were used to calculate odds ratios (ORs) and 95% confidence intervals (CIs). A *p*-value < 0.05 was considered statistically significant.

## 3. Results

### 3.1. Social Demographics of the Study Participants

A total of 1210 Schoolchildren from grades 1 to 12, including 48.4% (586/1210) boys and 51.6% (624/1210) girls, were enrolled. Out of the total enrolled subjects, 18.3% (222/1210) were confirmed as *S. aureus*, of which the MRSA proportion was 41% (91/222). Among the *S. aureus* isolates, the highest carriage rate (26.3%; 128/222) was detected in the 6- to 10-year age group. Age groups 11 to 14 and 15 to 18 were 14.4% (58/222) and 11.3% (36/222), respectively, Table 1. Among MRSA isolates, the 6- to 10-year age group showed a carriage rate of 48%, the 11- to 14-year age group 32.8%, and the 15- to 18-year age group was 27.8%. We did not find any difference based on the total number of household members. MRSA was detected more in males compared to females (49.5% vs. 33.3%, *p* = 0.015). Among the *S. aureus* carriage population, the antibiotic usage in the past month was associated with MRSA detection. Most schoolchildren attended public school (97.4%, *n* = 1178) and only a few were attending private schools (2.6%, *n* = 32). Type of school attendance and *S. aureus* carriage did not show any difference, Table 2.

### 3.2. Antibiotic Susceptibility Patterns

**Figure 1:**
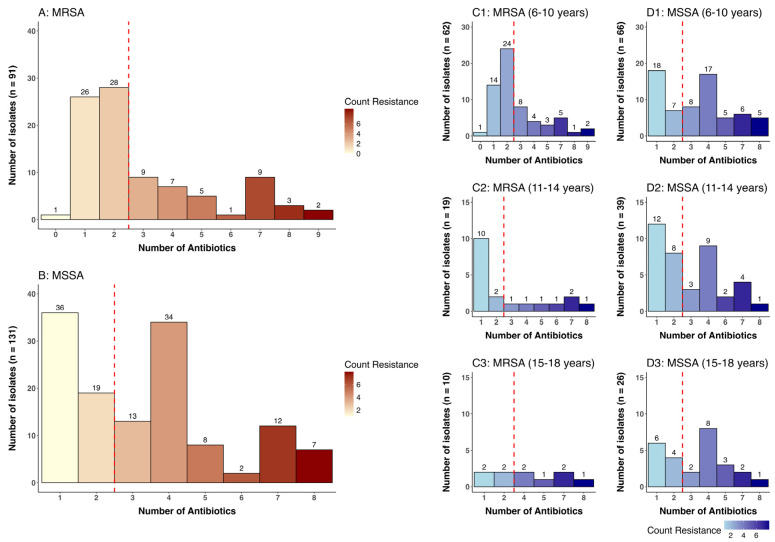
Distribution of multi-drug resistance among MRSA and MSSA isolates by age groups. Key: Number of isolates to more than 3 antibiotics; *MRSA* = A1 (15 isolates), A2 (6 isolates), and A3 (6 isolates). *MSSA* = B1 (34 isolates), B2 (16 isolates), and B3 (16 isolates). Abbreviations: MRSA = Methicillin-resistant *Staphylococcus aureus*, MSSA = Methicillin-susceptible *Staphylococcus aureus*.

Antibiotic susceptibility testing revealed high levels of resistance among *S. aureus* isolates to trimethoprim-sulfamethoxazole (100%), erythromycin (65.3%), and clindamycin (43%). Among MRSA isolates, resistance was detected to trimethoprim-sulfamethoxazole (100%), erythromycin (68.2%), and clindamycin (45.1%), as can be seen in Table 3. Multidrug resistance (MDR), defined as resistance to more than three antibiotics, was 40% (36/91) among MRSA isolates and 50.4% (66/131) among MSSA isolates, Figure 1 MDR.

### 3.3. MecA and PVL Genes Detection

In this study, 41% (91/222) of *S. aureus* isolates exhibited the *mec*A gene, while 4% (8/222) harbored the pvl gene in two MRSA and six MSSA isolates. The presence of pvl genes has been reported to be epidemiologically linked with CA-MRSA strains that contain SCC*mec* type IV, V, VI, VII, and VIII [23].

### 3.4. SCCmec Distribution for MRSA

**Figure 2:**
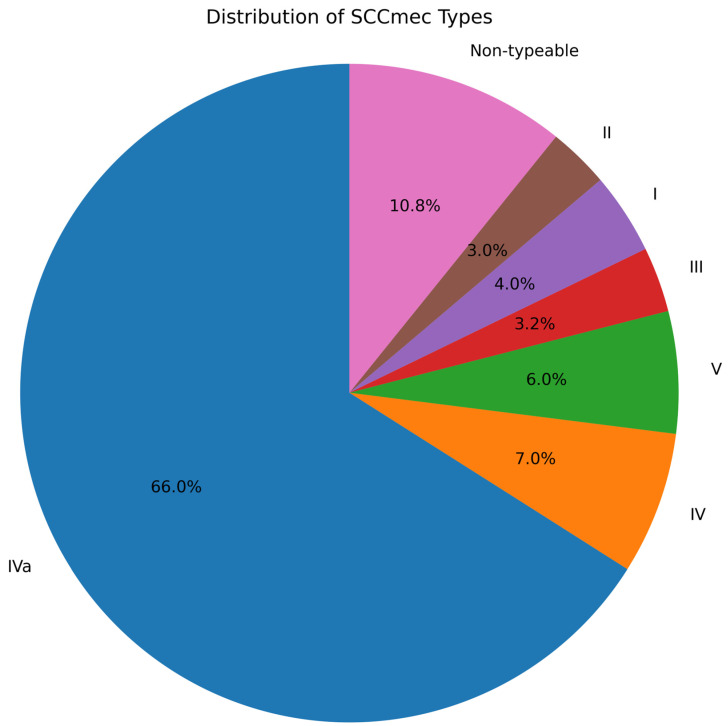
*SCCmec* distribution among MRSA isolates (*n* = 91). KEY: *SCCmec* type IVa demonstrated to be dominant (66.0%), followed by subtype IVa (7.0%) and type V (6.0%), all of which are associated with community MRSA.

To further characterize the MRSA isolates, SCC*mec* distribution was analyzed. SCC*mec* type IVa was demonstrated to be dominant. SCC*mec* typing of the 91 MRSA isolates revealed the following distribution: IVa 66.0% (60/91), IV 7.0% (6/91), V 6.0% (5/91), III 3.2% (3/91), I 4.0% (4/91), II 3.0% (3/91), and non-typeable 10.8% (10/91). SCC*mec* type IVa was dominant, accounting for two-thirds of isolates and indicating a predominance of community-associated SCCmec elements in this population Figure 2.

### 3.5. MLST of S. aureus Isolates

**Figure 3:**
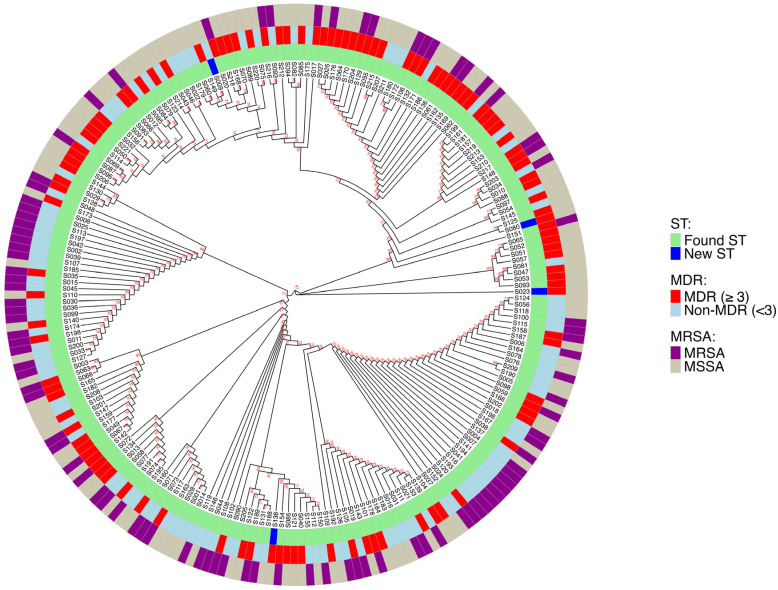
Phylogenetic analysis and MLST. KEY: Multi-locus strain types were computationally mapped onto the phylogenetic tree. The colored inner ring denotes ST(s), the second ring denotes MDR, and the outer ring denotes MRSA/MSSA. Abbreviations: ST = Sequence Type (s), MDR = multi-drug resistant, MRSA = Methicillin-resistant *Staphylococcus aureus*, MSSA = Methicillin-susceptible *Staphylococcus aureus*.

Overall, 53 different STs were identified from all 222 *S. aureus* isolates. The most prevalent STs identified by MLST were ST45 (18.0%), ST546 (10.8%), and ST188 (7.2%), accounting for 36% of the total isolates. Among the MSSA isolates, ST 45, ST546, and ST 188 were the most prevalent, accounting for 29%. The same ST45, ST546, and ST188 accounted for 50% of MRSA isolates. All *S. aureus* isolates were assigned to different CCs; the dominant CCs were CC45 (53.6%) and CC1 (22.5%), accounting for 76.1% of all the isolates. A subset of isolates remained untypeable by MLST. All ST types were correlated with MRSA and MDR Figure 3. The multidrug resistance nature of *S. aureus* isolates at Nha Trang, Vietnam are described in Table 4.

## 4. Discussion

This study targeted school children from three educational stages from four communes in Nha Trang, Vietnam, representing a community surveillance study to investigate the prevalence of *S. aureus* nasopharyngeal colonization, related antibiotic susceptibility, and molecular characteristics of MRSA. Among 1210 samples collected, 222 (18.3%) were positive for *S. aureus*, of which 91 (41.0%) were MRSA (*mec*A-positive), corresponding to an overall MRSA prevalence of 7.5% across all samples. The detection of MRSA in an apparently healthy, school-going population underscores the ongoing shift of MRSA from a predominantly hospital-acquired pathogen to one increasingly established within community settings. The prevalence of MRSA carriage among school children aged 6–10 years was 48% compared with the 11–14-year group (32.8%), and 27.8% for the 15–18-year age group. This suggests that younger children may represent an important reservoir for community transmission. Behavioral and biological factors, such as immature immunity, close contact in crowded classrooms, and limited adherence to hygiene practices, could account for this finding. Similar age-associated carriage trends have been reported in studies from other Asian settings, supporting the need for early school-based interventions. This is consistent with global reports documenting the emergence and spread of MRSA in school environments, households, and other non-healthcare populations [24]. The carriage rates declined in older age groups, a trend that has been reported in other studies. Antimicrobial susceptibility testing and molecular typing were performed on all 222 *S. aureus* isolates. While 40% (36/91 MRSA) exhibited resistance to three or more classes of antibiotics, indicating a high level of multidrug resistance, all isolates were susceptible to vancomycin, quinupristin/dalfopristin, linezolid, and daptomycin, confirming that these antibiotics continue to be effective therapeutic options for MRSA infections.

SCC*mec* typing of the 91 MRSA isolates revealed that IVa 66.0% (60/91) was the predominant type, followed by IV 7.0% (6/91), V 6.0% (5/91), III 3.2% (3/91), I 4.0% (4/91), II 3.0% (3/91), and non-typeable 10.8% (10/91). Our result indicated that SCC*mec* type IVa was the major type, accounting for two-thirds of isolates and indicating a predominance of community-associated *SCC*mec elements among the study participants.

Asymptomatic colonization of *S. aureus* increases the risk of invasive infections, particularly in school children. Although colonization itself may not cause immediate harm, it serves as a reservoir for potential horizontal transmission within communities. Studies have demonstrated that individuals colonized at the anterior nares are at heightened risk for several infections, including pneumonia.

In the current study, 18.3% of nasal specimens tested positive for *S. aureus;* of these, 41% isolates were MRSA, higher than a study conducted by Islam S et al. Overall, 24 out of 234 samples from children under ten years of age included *S. aureus*, according to another cross-sectional investigation; children between the ages of eight and ten had the highest carriage rate, 14.3% [25]. In our study, *S. aureus* carriage between male and female children did not show any significant difference, unlike a study conducted by Islam et al., in which *S. aureus* carriage was significantly more prevalent in male children than in female children. The differences in carriage rates and epidemiological characteristics may be due to the differences in socioeconomic, behavioral, and other risk factors in different populations at different study sites. Several investigations found that between 20 and 46 percent of school-aged children have *S. aureus* resistance to erythromycin. Less than 40% of isolates were susceptible to erythromycin and azithromycin, according to a study done in Bangladesh. In contrast, this study found that *S. aureus* displayed higher macrolide resistance, with 65.2% resistant to erythromycin

The proportion of PVL genes among MRSA strains in this study was low, 8%, aligning with findings previously reported in Japan. In contrast, a significantly higher PVL prevalence (over 48%) has been reported in countries such as Colombia, India, and Saudi Arabia [23]. This suggests that the prevalence of PVL may vary based on geographical location. The presence of PVL toxin genes cannot be considered a definitive marker of CA-MRSA, as these genes may also occur in HA-MRSA or be absent in certain CA-MRSA strains. Although highly virulent CA-MRSA strains carrying PVL genes are known to prevail in the world [26]. CA-MRSA pathogenesis has been linked to PVL, which is produced by most CA-MRSA isolates; however, the absence of PVL is no proof against community origin. This was documented in Australia, Japan, and China, where most CA-MRSA isolates lack the PVL gene [27]. ST45, ST546, and ST188 were the most predominant STs in this study. CC45 dominated 53.6%, followed by CC1 22.5% accounting for 76.1% of all the isolates. The successful persistence of the ST45–SCCmec IVa lineage among school-aged children, despite the low prevalence of the Panton–Valentine leukocidin (PVL) gene, suggests that factors other than classical virulence determinants may drive its transmission and stability in this population. ST45 is a globally distributed lineage that has been associated with efficient colonization rather than overt virulence, indicating that enhanced nasal fitness, host adaptation, and effective person-to-person transmission may contribute to its success. The presence of SCCmec IVa, a relatively small and mobile genetic element, may impose a lower fitness cost compared to larger SCCmec types, thereby facilitating stable maintenance and spreading in community settings. In school environments characterized by frequent close contact, shared surfaces, and repeated exposure, colonization efficiency and persistence may be more advantageous than high virulence. Additionally, non-PVL virulence factors, biofilm formation capacity, and immune evasion mechanisms may play important roles in sustaining carriage within this age group.

This study revealed several important findings. First, the MRSA Carriage rate varied notably across age groups, with younger participants exhibiting higher MRSA prevalence. Two MRSA isolates from children aged 6–10 years exhibited resistance to nine antibiotics, while one isolate from a participant aged 11–14 years and another from a participant aged 15–18 years each showed resistance to eight antibiotics. This age-associated pattern may be attributed to differences in exposure risk or developmental changes in immune status among school-aged children. Second, MSSA carriers showed higher resistance to a variety of antibiotics in comparison to MRSA carriers, particularly beta-lactams, macrolides, and fluoroquinolones.

Third, some isolates were non-typeable by MLST. This likely reflects the broader genetic diversity of carriage strains in healthy schoolchildren compared with the more clonal populations typically observed in clinical isolates. Many of these lineages are likely to be novel or regionally restricted and thus not yet represented in the MLST database. In addition, mixed colonization and occasional incomplete allele profiles may further contribute to unassigned STs in this population; further characterization using higher-resolution approaches, such as whole-genome sequencing, would be valuable in future studies to fully elucidate their genetic backgrounds. Finally, all isolates exhibited susceptibility to vancomycin, linezolid, and daptomycin, supporting their continued effectiveness for treating severe MRSA infections.

Future research should focus on comprehensive molecular characterization, particularly using next-generation sequencing of *S. aureus* and MRSA isolates, and explore environmental and animal reservoirs through a one health approach to more fully understand transmission dynamics of MRSA strains in Vietnam. Regular screening and monitoring of nasal carriage can provide critical data to inform targeted interventions. Such proactive measures not only help protect school communities but also support broader public health efforts to control the spread of antimicrobial-resistant pathogens in the communities.

Study limitation: This cross-sectional, community-based survey was conducted, and data were collected at a single point in time; data and samples collected may limit the generalizability of the findings across different seasons in Nha Trang, Vietnam.

## 5. Conclusions

In summary, the majority of MRSA isolates identified in this study demonstrated resistance to varying classes of non-β-lactam antibiotics, but all the isolates were sensitive to vancomycin and linezolid, ensuring their clinical application. Continuous molecular surveillance of MRSA in School children is essential to monitor antimicrobial resistance patterns, clonal evolution, emerging community reservoirs, and guide targeted infection control strategies aimed at mitigating MRSA spread across the population in Nha Trang, Vietnam. This study will be useful for future preventive measures against MRSA in the community.

## Figures and Tables

**Table 1:** Demographic and characteristics of the study participants *(n* = 1210), [*S. aureus* = 18.3%, 222)].

Variable	Positive (*N* = 222 ^1^)	Negative (*N* = 988 ^1^)	Univariable Regression		
			OR	95% CI	*p*-Value
Sex					
Male	105 (17.9)	481 (82.1)	Ref	Ref	
Female	117 (18.8)	507 (81.3)	1.06	0.79–1.42	0.709
Age group (years)					
6–10	128 (26.3)	359 (73.7)	Ref	Ref	
11–14	58 (14.4)	345 (85.6)	0.47	0.33–0.66	<0.001
15–18	36 (11.3)	284 (88.8)	0.36	0.24–0.53	<0.001
Antibiotic use in the last month					
Yes	12 (18.2)	54 (81.8)	Ref	Ref	
No	194 (18.0)	886 (82.0)	0.99	0.53–1.96	0.964
Took medicine, but do not know	16 (25.0)	48 (75.0)	1.5	0.65–3.55	0.346
Education level					
Primary school	132 (26.0)	376 (74.0)	Ref	Ref	
Secondary school	56 (13.9)	346 (86.1)	0.46	0.32–0.65	<0.001
High school	34 (11.3)	266 (88.7)	0.36	0.24–0.54	<0.001
School type					
Public	220 (18.7)	958 (81.3)	Ref	Ref	
Private	2 (6.3)	30 (93.8)	0.29	0.05–0.97	0.092
Class population					
Mean (Min–Max)	40.5 (32.0–52.0)	40.8 (24.0–69.0)	1.06	0.91–1.22	0.479
No of household members living together					
2	5 (13.5)	32 (86.5)	Ref	Ref	
3	31 (14.5)	183 (85.5)	1.08	0.42–3.36	0.876
4	139 (20.6)	535 (79.4)	1.66	0.69–4.93	0.3
5	27 (14.9)	154 (85.1)	1.12	0.43–3.50	0.826
6	15 (18.8)	65 (81.3)	1.48	0.52–4.86	0.486
>7	5 (20.8)	19 (79.2)	1.68	0.42–6.81	0.454

^1^ *N = number* (%); Class population = Mean (Min–Max). Abbreviations: *N* = Total number of isolates, CI = Confidence Interval, OR = Odds Ratio.

**Table 2:** Demographics and characteristics of MRSA (41%, 91/222) isolates.

Variable	MRSA (N = 91 ^1^)	MSSA (N = 131 ^1^)	Univariable Regression		
			OR	95% CI	*p*-Value
Sex					
Male	52 (49.5)	53 (50.5)	Ref	Ref	
Female	39 (33.3)	78 (66.7)	0.51	0.29–0.87	0.015
Age group (years)					
6–10	62 (48.4)	66 (51.6)	Ref	Ref	
11–14	19 (32.8)	39 (67.2)	0.52	0.27–0.98	0.047
15–18	10 (27.8)	26 (72.2)	0.41	0.18–0.89	0.030
Antibiotic use in the last month					
Yes	9 (75.0)	3 (25.0)	Ref	Ref	
No	75 (38.7)	119 (61.3)	0.21	0.05–0.73	0.022
Took medicine, but I do not know	7 (43.8)	9 (56.3)	0.26	0.04–1.25	0.106
School level					
Primary school	63 (47.7)	69 (52.3)	Ref	Ref	
Secondary school	18 (32.1)	38 (67.9)	0.52	0.26–0.99	0.050
High school	10 (29.4)	24 (70.6)	0.46	0.19–1.00	0.059
School type					
Public	91 (41.4)	129 (58.6)	Ref	Ref	
Private	0 (0.0)	2 (100.0)	0	0	0.988
Class population					
Mean (Min–Max)	40.7 (32.0–52.0)	40.4 (38.0–42.0)	1.02	0.95–1.09	0.673
No of household members living together					
2	2 (40.0)	32 (86.5)	Ref	Ref	
3	14 (45.2)	17 (54.8)	1.24	0.18–10.4	0.830
4	53 (38.1)	86 (61.9)	0.92	0.15–7.19	0.933
5	14 (51.9)	13 (48.1)	1.62	0.23–13.8	0.628
6	4 (26.7)	11 (73.3)	0.55	0.06–5.30	0.576
>7	4 (80.0)	1 (20.0)	6.55	0.43–178	0.214

^1^ N = number (%); Class population = Mean (Min–Max). Abbreviations: N = Total number of isolates, CI = Confidence Interval, OR = Odds Ratio.

**Table 3:** Antimicrobial resistance pattern of *S. aureus*, MRSA, and MSSA isolates.

Antibiotic	*S. aureus* N = 220		MRSA N = 90		MSSA N = 130		
	R, N (%)	S, N (%)	R, N (%)	S, N (%)	R, N (%)	S, N (%)	*p*-Value
SXT	220 (100.0)	0 (0.0)	90 (100.0)	0 (0.0)	130 (100.0)	0 (0.0)	N/A
Erythromycin	143 (65.0)	77 (35.0)	62 (68.9)	28 (31.1)	81 (62.3)	49 (37.7)	0.3
Clindamycin	94 (42.7)	126 (57.3)	36 (40.0)	54 (60.0)	54 (44.6)	72 (55.4)	0.5
Gentamicin	84 (38.2)	136 (61.8)	21 (23.3)	69 (76.7)	63 (48.5)	67 (51.5)	0.001
Tetracycline	39 (17.7)	181 (82.3)	12 (13.3)	78 (86.7)	27 (20.8)	103 (79.2)	0.2
Moxifloxacin	38 (17.3)	182 (82.7)	15 (16.7)	75 (83.3)	23 (17.7)	107 (82.3)	0.8
Ciprofloxacin	37 (16.8)	183 (83.2)	15 (16.7)	75 (83.3)	22 (16.9)	108 (83.3)	0.9
Levofloxacin	37 (16.8)	183 (83.2)	15 (16.7)	75 (83.3)	22 (16.9)	108 (83.1)	0.9
Ampicillin	18 (8.2)	101 (45.9)	7 (7.8)	21 (23.3)	11 (8.5)	80 (61.5)	0.001
Daptomycin	3 (1.4)	217 (98.6)	1 (1.1)	89 (98.9)	2 (1.5)	128 (98.5)	0.9
Imipenem	0 (0.0)	219 (99.5)	1 (1.1)	89 (98.9)	0 (0.0)	129 (99.2)	0.9
Rifampicin	0 (0.0)	0 (0.0)	0 (0.0)	90 (100.0)	0 (0.0)	0 (0.0)	0.084
QDA	0 (0.0)	0 (0.0)	0 (0.0)	0 (0.0)	0 (0.0)	0 (0.0)	N/A
Linezolid	0 (0.0)	0 (0.0)	0 (0.0)	0 (0.0)	0 (0.0)	0 (0.0)	N/A
Vancomycin	0 (0.0)	0 (0.0)	0 (0.0)	0 (0.0)	0 (0.0)	0 (0.0)	N/A
Tigecycline	0 (0.0)	0 (0.0)	0 (0.0)	0 (0.0)	0 (0.0)	0 (0.0)	N/A
Nitrofurantoin	0 (0.0)	0 (0.0)	0 (0.0)	0 (0.0)	0 (0.0)	0 (0.0)	N/A

Key: N = Total number of isolates, S = Sensitive, R = Resistant, QDA = Quinupristin/Dalfopristin, TMP = Trimethoprim/Sulfamethoxazole, MRSA = Methicillin-resistant *Staphylococcus aureus*, MSSA = Methicillin-susceptible *Staphylococcus aureus*, N/A = Not applicable. Note: Two isolates did not have AST results.

**Table 4:** Multidrug-resistant *Staphylococcus aureus* isolates exhibited resistance to 3–9 antibiotics, with patterns varying by sequence type.

No of Antibiotics	Multi-Drug-Resistant Pattern	No of Resistance Strains (*n* = 90)		
		No	%	ST Type
3	ERY, CLI, SXT	16	(14.4)	45,278, 546, 617, 1774
	GEN, TCY, SXT	3	(2.7)	7, 6954
	CIP, LVX, SXT	1	(0.9)	188
	ERY, SXT, AMP	1	(0.9)	45
	ERY, TCY, SXT	1	(0.9)	1906
4	GEN, ERY, CLI, SXT	23	(20.7)	1, 14, 45, 278, 296, 320
	ERY, CLI, TCY, SXT	8	(7.2)	45, 199, 610, 617, 1232
	GEN, ERY, TCY, SXT	8	(7.2)	14, 15, 278, 320, 1659
	ERY, CLI, SXT, AMP	1	(0.9)	45
	GEN, CLI, LVX, SXT	1	(0.9)	2372
5	GEN, ERY, CLI, TCY, SXT	6	(5.4)	88, 264, 319, 411, 2261
	GEN, ERY, CLI, SXT, AMP	3	(2.7)	320, 411
	ERY, CLI, TCY, SXT, AMP	2	(1.8)	400, 567
	GEN, CIP, LVX, MFX, SXT	1	(0.9)	8141
	GEN, ERY, CLI, SXT, DAP	1	(0.9)	45
6	GEN, CIP, LVX, ERY, CLI, SXT	1	(0.9)	2201
	GEN, CIP, LVX, ERY, TCY, SXT	1	(0.9)	320
	GEN, ERY, CLI, TCY, SXT, AMP	1	(0.9)	188
7	GEN, CIP, LVX, MFX, ERY, CLI, SXT	18	(16.2)	148, 188, 296, 411, 617
	CIP, LVX, MFX, ERY, CLI, TCY, SXT	1	(0.9)	2655
	GEN, CIP, LVX, ERY, CLI, SXT, AMP	1	(0.9)	1054
	GEN, CIP, LVX, ERY, CLI, TCY, SXT	1	(0.9)	546
8	GEN, CIP, LVX, MFX, ERY, CLI, SXT, AMP	7	(6.3)	188, 296, 567, 617
	GEN, CIP, LVX, MFX, ERY, CLI, TCY, SXT	2	(1.8)	188, 2139
9	GEN, CIP, LVX, MFX, ERY, CLI, TCY, SXT, AMP	2	(1.8)	6775

Abbreviations: ERY = Erythromycin, CLI = Ciprofloxacin, SXT = Trimethoprim-Sulfamethoxazole, GEN = Gentamycin, TCY = Tetracycline, LVX = Levofloxacin, AMP = Ampicillin, MFX = Moxifloxacin, DAP = Daptomycin.

## Data Availability

The data that support the findings of this study are described in the Supplementary Material; further inquiries can be directed to the corresponding author.

## Supplementary Materials

The following supporting information can be downloaded at: https://www.mdpi.com/article/10.3390/pathogens15020238/s1, Table S1: Primers used for SCC*mec* typing. Table S2: MLST primers for 7 housekeeping genes. Table S3: MDR patterns across age groups. Table S4: MLST data. Table S5: MDR in relation to STs. Figure S1: Antibiotic Susceptibility pattern of MRSA and MSSA isolates. Figure S2: Prevalence of *mecA* and PVL gene. Figure S3: eBURST Visualization results.

## Abbreviations

The following abbreviations are used in this manuscript.

AST	Antimicrobial Susceptibility Test
ABR	Antibiotic Resistance
MDR	Multidrug Resistance
MRSA	Methicillin-resistant *Staphylococcus aureus*
CA-MRSA	Community-acquired Methicillin-resistant *Staphylococcus aureus*
PCR	Polymerase Chain Reaction
MIC	Minimum Inhibitory Concentration
MLST	Multi-Locus Sequence Typing
NIHE	National Institute of Hygiene and Epidemiology
KEMRI	Kenya Medical Research Institute
QDA	Quinupristin/Dalfopristin
SXT	Sulfamethoxazole-Trimethoprim
SBA	Sheep Blood Agar
MSA	Mannitol Salt Agar
PBP	Penicillin-binding Protein
MSSA	Methicillin-sensitive *S. aureus*
CLSI	Clinical and Laboratory Standards Institute
PVL	Panton–Valentine Leukocidin
DNA	Deoxyribonucleic Acid
SCC*mec*	*Staphylococcus* Cassette Chromosome *mec*
Cls	Confidence Intervals
ORs	Odd Ratios
ATCC	American Type Culture Collection
ST	Sequence Type
USA	United States of America
NUC	Nuclease gene
STGG	Skim Milk–Tryptone–Glucose–Glycerol
NU	Nagasaki University
PID	Pediatric Infectious Diseases
