# Supplementary material for: Prevalence, Antimicrobial Resistance Profiles, and Molecular Characteristics of Methicillin-Resistant Staphylococcus aureus Among School Children in Nha Trang, Central Vietnam
Source: Pathogens. 2026 Feb 22;15(2):238. doi: 10.3390/pathogens15020238 (PMC12943241; doi:10.3390/pathogens15020238)

Prevalence, Antimicrobial Resistance Profiles and Molecular Characteristics of Methicillin-Resistant S*taphylococcus aureus* Among School Children in Nha Trang, Central Vietnam

Stephen Anyona Omae[1,2,3], Shah Mohammad Monir[1], Hien-Anh Thi Nguyen[4], Kim-Mai Huynh[5], Natsuki Ariyoshi[1], Liên Thuy Le[5], Dat Thanh Le[5], Trieu Bao Nguyen[5], Hoang Huy Le[3], Luong Dinh Nguyen[1,2,5], Miyuki Tsuruoka[1], Hirono Otomaru[1], Erik Koehne[1], Michiko Toizumi[1,6], Duc-Anh Dang[4], Hung Do Thai[1,5], Lay-Myint Yoshida[1,2,6] *

Supplementary material.

Table S1: Primers used for SCCmec gene amplification 2
Table S2: Primers used for MLST 3
Table S3: Multidrug Resistance Patterns of MRSA and MSSA isolates across different age groups. 5
Table S4: Result of MLST 12
Table S5: Multidrug Resistance Patterns of MRSA and MSSA isolates across different ST 19

Figure S1: Prevalence of mecA against PVL gene 4
Figure S2: Antibiotic susceptibility pattern of MRSA and MSSA isolates 11
Figure S3: Visualize associated isolate data on the goeBURST image 26

Table S1: Primers used for SCCmec gene amplification

Gene	Primer	Oligonucleotide sequence 5'➔3'	Amplicon size	Specificity	Reference
SCCmec	Type I-F	GCTTTAAAGAGTGTCGTTACAGG	**613**	**SCCmec I**	**[61]**
	Type I-R	GTTCTCTCATAGTATGACGTCC			
	Type II-F	CGTTGAAGATGATGAAGCG	**398**	**SCCmec II**	**[61]**
	Type II-R	CGAAATCAATGGTTAATGGACC			
	Type II-F2	TAGCTTATGGTGCTTATGCG	**128**	**SCCmec II**	**[61]**
	Type II-R2	GTGCATGATTTCATTTGTGGC			
	Type III-F	CCATATTGTGTACGATGCG	**280**	**SCCmec III and IIIA**	**[61]**
	Type III-R	CCTTAGTTGTCGTAACAGATCG			
	Type III-F5	TTCTCATTGATGCTGAAGCC	**257**	**SCCmec III and IIIA**	**[61]**
	Type III-R6	GTGTAATTTCTTTTGAAAGATATGG			
	Type-IVa-F	GCCTTATTCGAAGAAACCG	**776**	**SCCmec IVa**	**[61]**
	Type-IVa-R	CTACTCTTCTGAAAAGCGTCG			
	Type-IVb-F	TCTGGAATTACTTCAGCTGC	**493**	**SCCmec IVb, IVF**	**[61]**
	Type-IVb-R	AAACAATATTGCTCTCCCTC			
	Type-IVc-F2	CCTGAATCTAAAGAGATACACCG	**200**	**SCCmec IVc, IVE**	**[61]**
	Type-IVc-R2	GGTTATTTTCATAGTGAATCGC			
	TypeIVd-F5	CTCAAAATACGGACCCCAATACA	**881**	**SCCmec IVd**	**[61]**
	TypeIVd-R6	TGCTCCAGTAATTGCTAAAG			
	TypeIVE-F3	CAGATTCATCATTTCAAAGGC	**175**	**SCCmec IVE, IVF**	**[61]**
	TypeIVE-F4	AACAACTATTAGATAATTTCCG			
	TypeV-F	GAACATTGTTACTTAAATGAGCG	**325**	**SCCmec V**	**[61]**
	TypeV-R	TGAAAGTTGTACCCTTGACACC			

Table S2: Primers used for MLST

Gene	Primer	Sequence (5` - 3`)	Base pair size	Reference
Carbamate kinase (arcC)	*arc*C-Up	TTGATTCACCAGCGCGTATTGTC	570bp	[41]
	*arc*C-Dn	AGGTATCTGCTTCAATCAGCG		
Shikimate dehydro genase (aroE)	*aro*E-Up	ATCGGAAATCCTATTTCACATTC	536bp	[41]
	*aro*E-Dn	GGTGTTGTATTAATAACGATATC		
Glycerol kinase (glpF)	*glp*F-Up	CTAGGAACTGCAATCTTAATCC	576bp	[41]
	*glp*F-Dn	TGGTAAAATCGCATGTCCAATTC		
Guanylate kinase (gmk)	*gmk*-Up	ATCGTTTTATCGGGACCATC	488bp	[41]
	gmk-Dn	TCATTAACTACAACGTAATCGTA		
Phosphate acetyltrans ferase (pta)	*pta*-Up	GTTAAAATCGTATTACCTGAAGG	575bp	[41]
	pta-Dn	GACCCTTTTGTTGAAAAGCTTAA		
Triosephosphate isomerase (tpi)	*tpi*-Up	TCGTTCATTCTGAACGTCGTGAA	475bp	[41]
	tpi-Dn	TTTGCACCTTCTAACAATTGTAC		
Acetyl coenzyme A ace tyltransferase (yqiL)	*yqi*L-Up	CAGCATACAGGACACCTATTGGC	598bp	[41]
	*yqi*L-Dn	CGTTGAGGAATCGATACTGGAAC		

Figure S1: Prevalence of *mecA* against PVL gene

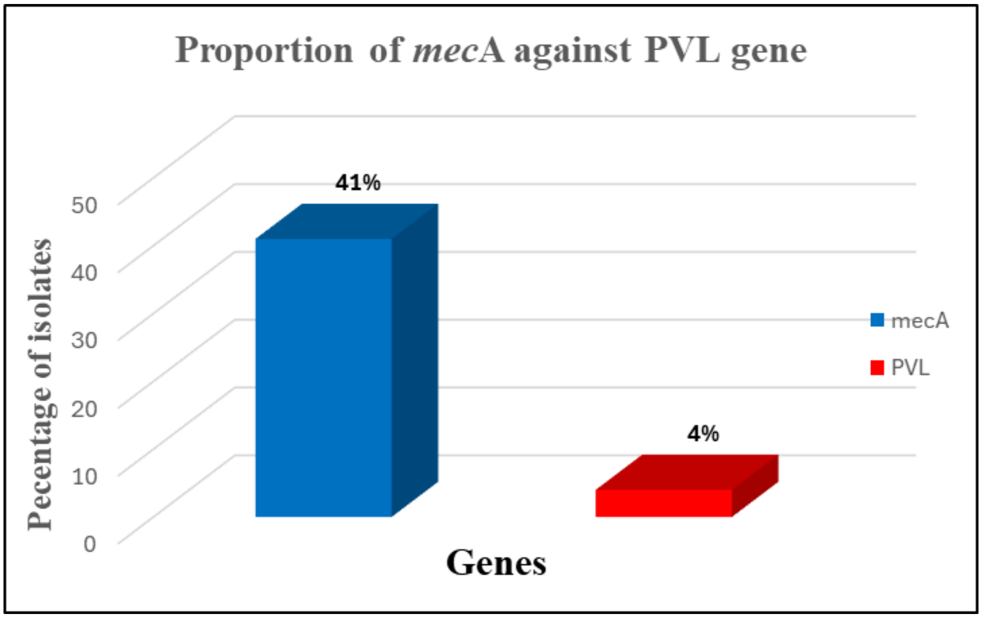

In this study 41% (91/222) of *S. aureus* isolates carried the *mec*A gene, while 4% (8/222, (2 MRSA isolates and 6 MSSA) harbored the pvl gene. The presence of PVL genes has been reported to be epidemiologically linked with CA-MRSA strains that contain SCC*mec* type IV, V, VI, VII and VIII

Table S3: Multidrug Resistance Patterns of MRSA and MSSA isolates across different age groups.

S. no	Group	Age bracket	Resistant antibiotics	No of resistant
1	MSSA	6-10	ERY, TMP	2
2	MRSA	6-10	TMP	1
3	MSSA	6-10	TMP	1
4	MRSA	6-10	ERY, TMP	2
5	MRSA	6-10	ERY, TMP	2
6	MRSA	6-10	ERY, TMP	2
7	MRSA	6-10	ERY, TMP	2
8	MRSA	6-10	TMP	1
9	MSSA	6-10	TMP	1
10	MSSA	6-10	GEN, ERY, CLI, TMP	4
11	MRSA	6-10	TMP	1
12	MRSA	11-14	GEN, ERY, CLI, TCY, TMP	5
13	MSSA	11-14	GEN, ERY, CLI, TMP	4
14	MSSA	11-14	TMP	1
15	MRSA	11-14	TMP	1
16	MSSA	11-14	TMP	1
17	MRSA	11-14	GEN, CIP, LVX, MFX, ERY, CLI, TMP	7
18	MRSA	11-14	TMP	1
19	MRSA	11-14	TMP	1
20	MRSA	15-18	GEN, CIP, LVX, MFX, ERY, CLI, TCY, TMP	8
21	MSSA	15-18	GEN, ERY, CLI, TMP	4
22	MSSA	15-18	TMP	1
23	MSSA	15-18	GEN, ERY, CLI, TMP	4
24	MSSA	15-18	GEN, CIP, LVX, MFX, ERY, CLI, TCY, TMP	8
25	MRSA	6-10	ERY, CLI, TMP	3
26	MRSA	6-10	ERY, TMP	2
27	MRSA	6-10	GEN, CIP, LVX, MFX, ERY, CLI, TMP	7
28	MSSA	6-10	GEN, CIP, LVX, MFX, ERY, CLI, TMP	7
29	MSSA	6-10	TMP	1
30	MSSA	6-10	GEN, ERY, CLI, TMP	4
31	MRSA	6-10	ERY, TMP	2
32	MRSA	6-10	TMP	1
33	MSSA	6-10	GEN, ERY, CLI, TMP	4
34	MRSA	6-10	ERY, TMP	2
35	MRSA	6-10	ERY, TMP	2
36	MRSA	6-10	ERY, CLI, TMP	3

S. no	Group	Age bracket	Resistant antibiotics	No of resistant
37	MRSA	6-10		0
38	MSSA	6-10	TMP	1
39	MRSA	6-10	ERY, CLI, TMP	3
40	MRSA	6-10	ERY, TMP	2
41	MSSA	6-10	GEN, ERY, CLI, TCY, TMP	5
42	MRSA	6-10	ERY, TMP	2
43	MRSA	6-10	ERY, TMP	2
44	MSSA	6-10	GEN, ERY, TCY, TMP	4
45	MRSA	6-10	TMP	1
46	MRSA	6-10	TMP	1
47	MSSA	6-10	TMP	1
48	MSSA	6-10	GEN, ERY, CLI, TMP	4
49	MRSA	6-10	ERY, TMP	2
50	MSSA	6-10	ERY, CLI, TMP	3
51	MSSA	6-10	GEN, ERY, TCY, TMP	4
52	MSSA	6-10	GEN, ERY, CLI, TCY, TMP	5
53	MSSA	6-10	GEN, ERY, CLI, TMP	4
54	MSSA	6-10	GEN, ERY, CLI, TMP	4
55	MSSA	6-10	GEN, TCY, TMP	3
56	MSSA	6-10	GEN, CIP, LVX, MFX, ERY, CLI, TMP	7
57	MSSA	6-10	ERY, TMP	2
58	MSSA	6-10	GEN, ERY, CLI, TMP	4
59	MRSA	6-10	TMP	1
60	MRSA	6-10	ERY, CLI, TMP	3
61	MSSA	6-10	GEN, TMP	2
62	MRSA	6-10	GEN, CIP, LVX, MFX, ERY, CLI, TMP	7
63	MRSA	6-10	GEN, CIP, LVX, MFX, ERY, CLI, TMP	7
64	MRSA	6-10	ERY, CLI, TCY, TMP	4
65	MSSA	6-10	CIP, LVX, MFX, TMP	4
66	MSSA	6-10	GEN, ERY, CLI, TCY, TMP	5
67	MSSA	11-14	GEN, ERY, CLI, TMP	4
68	MRSA	6-10	ERY, CLI, TCY, TMP	4
69	MRSA	6-10	ERY, CLI, TCY, TMP	4
70	MSSA	6-10	TMP	1
71	MSSA	11-14	GEN, ERY, CLI, TMP	4
72	MRSA	6-10	TMP	1
73	MRSA	6-10	ERY, CLI, TMP	3
74	MRSA	6-10	ERY, TMP	2
75	MRSA	11-14	TMP	1
76	MSSA	11-14	TMP	1

S. no	Group	Age bracket	Resistant antibiotics	No of resistant
77	MRSA	11-14	TMP	1
78	MRSA	11-14	TMP	1
79	MSSA	11-14	ERY, TMP	2
80	MSSA	11-14	TMP	1
81	MSSA	11-14	GEN, CIP, LVX, MFX, ERY, CLI, TMP	7
82	MSSA	11-14	TMP	1
83	MSSA	11-14	TCY, TMP	2
84	MSSA	11-14	TMP	1
85	MSSA	11-14	ERY, CLI, TCY, TMP	4
86	MSSA	11-14	GEN, TCY, TMP	3
87	MSSA	11-14	GEN, CIP, LVX, MFX, ERY, CLI, TMP	7
88	MRSA	11-14	ERY, CLI, TCY, TMP	4
89	MSSA	15-18	ERY, CLI, TMP	3
90	MSSA	15-18	ERY, TMP	2
91	MRSA	15-18	ERY, TMP	2
92	MSSA	15-18	ERY, TMP	2
93	MRSA	15-18	TCY, TMP	2
94	MSSA	15-18	GEN, ERY, CLI, TMP	4
95	MSSA	15-18	GEN, ERY, CLI, TCY, TMP	5
96	MSSA	15-18	TMP	1
97	MSSA	15-18	GEN, ERY, TCY, TMP	4
98	MSSA	15-18	GEN, TMP	2
99	MSSA	15-18	GEN, ERY, CLI, TMP, DAP	5
100	MRSA	6-10	ERY, TMP	2
101	MRSA	6-10	ERY, TMP	2
102	MRSA	6-10	GEN, ERY, CLI, TMP, AMP	5
103	MRSA	6-10	ERY, CLI, TMP, AMP	4
104	MSSA	6-10	GEN, CIP, LVX, MFX, ERY, CLI, TMP, AMP	8
105	MSSA	6-10	ERY, CLI, TMP	3
106	MRSA	6-10	GEN, ERY, CLI, TMP, AMP	5
107	MRSA	6-10	GEN, CIP, LVX, MFX, ERY, CLI, TCY, TMP, AMP	9
108	MRSA	6-10	ERY, TMP	2
109	MRSA	6-10	ERY, TMP	2
110	MSSA	6-10	ERY, TMP, AMP	3
111	MSSA	6-10	GEN, ERY, CLI, TMP	4
112	MRSA	6-10	ERY, TMP	2
113	MSSA	6-10	TMP	1
114	MRSA	6-10	TMP	1
115	MSSA	6-10	GEN, ERY, TCY, TMP	4
116	MRSA	6-10	ERY, TMP	2

S. no	Group	Age bracket	Resistant antibiotics	No of resistant
117	MRSA	6-10	TMP	1
118	MRSA	6-10	GEN, ERY, CLI, TCY, TMP	5
119	MSSA	6-10	TMP	1
120	MRSA	6-10	TMP	1
121	MRSA	6-10	TMP	1
122	MSSA	6-10	GEN, ERY, CLI, TMP, AMP	5
123	MSSA	6-10	GEN, CIP, LVX, MFX, TMP	5
124	MSSA	6-10	GEN, CIP, LVX, MFX, TMP	5
125	MSSA	6-10	MFX, ERY, TMP	3
126	MSSA	6-10	GEN, CIP, LVX, MFX, ERY, CLI, TMP	7
127	MRSA	6-10	TMP	1
128	MRSA	6-10	TMP	1
129	MRSA	6-10	ERY, CLI, TMP	3
130	MRSA	6-10	GEN, CIP, LVX, MFX, ERY, CLI, TMP	7
131	MSSA	11-14	TMP	1
132	MRSA	11-14	TMP	1
133	MRSA	11-14	GEN, CIP, LVX, MFX, ERY, CLI, TCY, TMP	8
134	MRSA	11-14	ERY, TMP	2
135	MSSA	11-14	GEN, ERY, TCY, TMP	4
136	MSSA	11-14	GEN, CIP, LVX, MFX, ERY, CLI, TMP, AMP	8
137	MSSA	11-14	GEN, ERY, CLI, TCY, TMP, AMP	6
138	MSSA	11-14	TMP	1
139	MSSA	11-14	GEN, TCY, TMP	3
140	MSSA	11-14	GEN, ERY, CLI, TMP	4
141	MSSA	11-14	GEN, CIP, LVX, MFX, ERY, CLI, TCY, TMP	8
142	MRSA	11-14	TMP	1
143	MSSA	11-14	GEN, ERY, CLI, TMP	4
144	MSSA	11-14	GEN, CIP, LVX, MFX, ERY, TCY, TMP	7
145	MSSA	11-14	CIP, LVX, MFX, ERY, CLI, TCY, TMP	7
146	MSSA	11-14	GEN, TMP	2
147	MSSA	11-14	TMP	1
148	MRSA	11-14	GEN, CIP, LVX, MFX, ERY, CLI, TMP	7
149	MRSA	15-18	GEN, CIP, LVX, MFX, ERY, CLI, TMP	7
150	MSSA	15-18	GEN, CIP, LVX, MFX, ERY, CLI, TMP, AMP	8
151	MRSA	15-18	TMP	1
152	MRSA	15-18	GEN, ERY, CLI, TMP	4
153	MRSA	15-18	TMP	1
154	MSSA	15-18	GEN, ERY, CLI, TMP	4
155	MRSA	15-18	GEN, ERY, CLI, TMP	4
156	MRSA	6-10	ERY, TMP	2

S. no	Group	Age bracket	Resistant antibiotics	No of resistant
157	MRSA	6-10	ERY, TMP	2
158	MSSA	6-10	GEN, CIP, LVX, MFX, ERY, CLI, TMP	7
159	MRSA	6-10	ERY, CLI, TMP	3
160	MRSA	6-10	ERY, TMP	2
161	MSSA	6-10	GEN, ERY, CLI, TMP	4
162	MRSA	6-10	GEN, CIP, LVX, MFX, ERY, CLI, TMP, AMP	8
163	MRSA	6-10	GEN, CIP, LVX, MFX, ERY, CLI, TMP	7
164	MSSA	6-10	TMP	1
165	MSSA	6-10	TMP	1
166	MSSA	6-10	TMP	1
167	MSSA	6-10	ERY, CLI, TCY, TMP	4
168	MSSA	6-10	ERY, CLI, TMP	3
169	MSSA	6-10	TMP	1
170	MSSA	6-10	GEN, CIP, LVX, MFX, ERY, CLI, TMP, AMP	8
171	MSSA	6-10	GEN, CIP, LVX, MFX, ERY, CLI, TMP, AMP	8
172	MSSA	6-10	GEN, CIP, LVX, MFX, ERY, CLI, TMP	7
173	MSSA	6-10	TMP	1
174	MRSA	6-10	GEN, CIP, LVX, MFX, ERY, CLI, TCY, TMP, AMP	9
175	MSSA	6-10	ERY, CLI, TMP	3
176	MRSA	6-10	ERY, TMP	2
177	MSSA	6-10	TMP	1
178	MSSA	6-10	GEN, CIP, LVX, MFX, ERY, CLI, TMP, AMP	8
179	MSSA	6-10	GEN, ERY, CLI, TMP	4
180	MSSA	6-10	GEN, ERY, TCY, TMP	4
181	MSSA	6-10	TMP	1
182	MSSA	6-10	TMP	1
183	MSSA	6-10	GEN, CIP, LVX, MFX, ERY, CLI, TMP	7
184	MSSA	6-10	ERY, TMP	2
185	MSSA	6-10	ERY, TMP	2
186	MSSA	6-10	ERY, TMP	2
187	MSSA	6-10	GEN, CIP, LVX, MFX, ERY, CLI, TMP, AMP	8
188	MSSA	6-10	ERY, CLI, TCY, TMP	4
189	MSSA	11-14	TMP	1
190	MRSA	6-10	ERY, CLI, TMP	3
191	MSSA	6-10	TMP	1
192	MSSA	6-10	ERY, CLI, TMP	3
193	MSSA	6-10	TMP	1
194	MRSA	6-10	ERY, TMP	2
195	MRSA	11-14	TMP, DAP	2
196	MSSA	11-14	ERY, TMP	2

S. no	Group	Age bracket	Resistant antibiotics	No of resistant
197	MRSA	11-14	TMP	1
198	MRSA	11-14	TMP	1
199	MRSA	11-14	ERY, CLI, TMP	3
200	MSSA	11-14	TMP	1
201	MSSA	11-14	TMP, DAP	2
202	MSSA	11-14	TCY, TMP	2
203	MSSA	11-14	ERY, CLI, TMP	3
204	MSSA	11-14	GEN, ERY, CLI, TMP	4
205	MRSA	11-14	GEN, CIP, LVX, MFX, ERY, CLI, TMP	7
206	MSSA	15-18	TMP	1
207	MSSA	11-14	GEN, ERY, TCY, TMP	4
208	MSSA	11-14	ERY, TMP	2
209	MSSA	11-14	TMP	1
210	MSSA	11-14	ERY, TMP	2
211	MSSA	15-18	TMP	1
212	MSSA	15-18	TMP	1
213	MSSA	15-18	GEN, CIP, LVX, MFX, ERY, CLI, TMP	7
214	MSSA	15-18	GEN, TMP	2
215	MSSA	15-18	TMP	1
216	MSSA	15-18	GEN, ERY, TCY, TMP	4
217	MRSA	15-18	ERY, CLI, TCY, TMP, AMP	5
218	MRSA	15-18	GEN, CIP, LVX, MFX, ERY, CLI, TMP	7
219	MSSA	15-18	ERY, CLI, TCY, TMP, AMP	5
220	MSSA	15-18	GEN, ERY, CLI, TMP	4
221	MSSA	15-18	ERY, CLI, TCY, TMP	4
222	MSSA	15-18	ERY, TCY, TMP	3

Figure S2: Antibiotic susceptibility pattern of MRSA and MSSA isolates

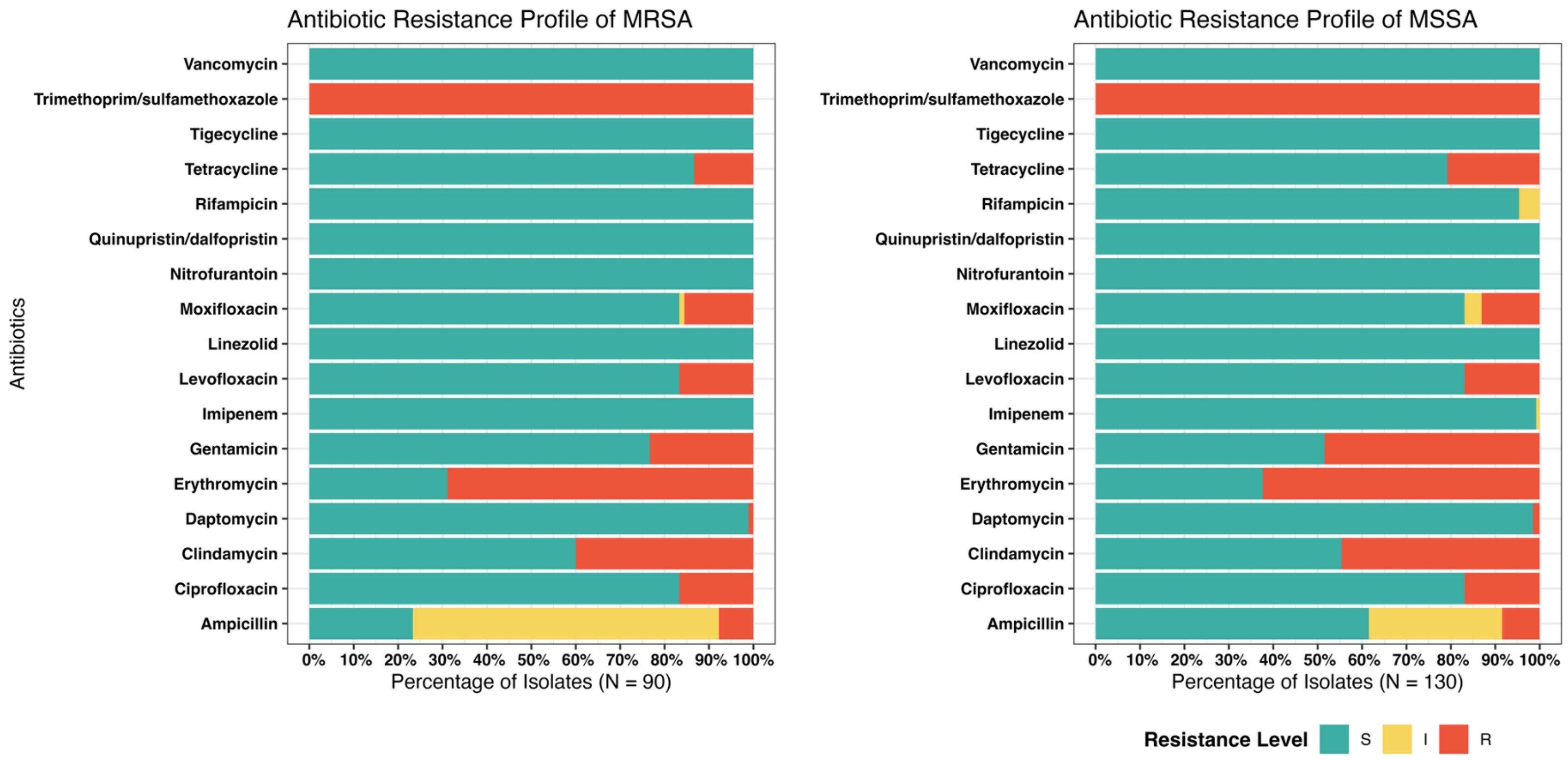

S=*Sensitive*, ***I***=*Intermediate* ***R***=*Resistant and* ***MSSA*** *= Methicillin Susceptible Staphylococcus aureus*

Table S4: Result of MLST

S. no	ST	arcC	aroE	glpF	gmk	pta	tpi	yqiL	clonal complex
S001	319	10	14	8	2	10	3	2	CC45
S002	546	10	14	1	6	10	3	2	CC45
S003	121	6	5	6	2	7	14	5	CC121
S004	45	10	14	8	6	10	3	2	CC45
S005	45	10	14	8	6	10	3	2	CC45
S006	45	10	14	8	6	10	3	2	CC45
S007	45	10	14	8	6	10	3	2	CC45
S008	546	10	14	1	6	10	3	2	CC45
S009	new_ST	3	1	8	1	1	1	1	
S010	1774	10	1	1	8	1	1	2	CC1
S011	546	10	14	1	6	10	3	2	CC45
S012	264	1	13	1	1	12	1	15	CC1
S013	278	1	14	8	6	10	3	2	CC45
S014	319	10	14	8	2	10	3	2	CC45
S015	546	10	14	1	6	10	3	2	CC45
S016	320	10	13	8	6	10	3	2	CC45
S017	2201	3	1	1	8	1	14	1	CC1
S018	45	10	14	8	6	10	3	2	CC45
S019	320	10	13	8	6	10	3	2	CC45
S020	2139	3	1	1	1	1	1	1	CC1
S021	320	10	13	8	6	10	3	2	CC45
S022	2125	1	1	1	1	1	1	3	CC1
S023	new_ST	10	14	8	34	10	3	2	
S024	45	10	14	8	6	10	3	2	CC45
S025	546	10	14	1	6	10	3	2	CC45
S026	188	3	1	1	8	1	1	1	CC1
S027	188	3	1	1	8	1	1	1	CC1
S028	319	10	14	8	2	10	3	2	CC45
S029	2655	1	14	1	6	10	3	2	CC45
S030	546	10	14	1	6	10	3	2	CC45
S031	320	10	13	8	6	10	3	2	CC45
S032	14	1	13	1	1	12	11	13	CC15
S033	546	10	14	1	6	10	3	2	CC45
S034	1774	10	1	1	8	1	1	2	CC1

S. no	ST	arcC	aroE	glpF	gmk	pta	tpi	yqiL	clonal complex
S035	546	10	14	1	6	10	3	2	CC45
S036	546	10	14	1	6	10	3	2	CC45
S037	45	10	14	8	6	10	3	2	CC45
S038	45	10	14	8	6	10	3	2	CC45
S039	546	10	14	1	6	10	3	2	CC45
S040	411	10	14	8	1	1	3	2	CC45
S041	45	10	14	8	6	10	3	2	CC45
S042	546	10	14	1	6	10	3	2	CC45
S043	1659	13	1	1	1	12	11	13	CC15
S044	45	10	14	8	6	10	3	2	CC45
S045	2231	10	14	1	1	10	3	2	CC45
S046	97	3	1	1	1	1	5	3	CC97
S047	2259	151	1	1	34	175	180	169	nan
S048	546	10	14	1	6	10	3	2	CC45
S049	617	3	14	8	6	10	3	2	CC45
S050	15	13	13	1	1	12	11	13	CC15
S051	2261	151	13	1	34	175	180	169	nan
S052	2261	151	13	1	34	175	180	169	nan
S053	2259	151	1	1	34	175	180	169	nan
S054	7	5	4	1	4	4	6	3	nan
S055	188	3	1	1	8	1	1	1	CC1
S056	45	10	14	8	6	10	3	2	CC45
S057	2261	151	13	1	34	175	180	169	nan
S058	278	1	14	8	6	10	3	2	CC45
S059	45	10	14	8	6	10	3	2	CC45
S060	805	3	3	1	1	1	1	1	CC1
S061	188	3	1	1	8	1	1	1	CC1
S062	188	3	1	1	8	1	1	1	CC1
S063	1232	3	35	167	2	20	26	39	nan
S064	188	3	1	1	8	1	1	1	CC1
S065	2261	151	13	1	34	175	180	169	nan
S066	1984	13	13	1	1	12	3	2	CC15
S067	617	3	14	8	6	10	3	2	CC45
S068	1232	3	35	167	2	20	26	39	nan
S069	2404	3	13	1	1	12	11	13	CC15

S. no	ST	arcC	aroE	glpF	gmk	pta	tpi	yqiL	clonal complex
S070	1	1	1	1	1	1	1	1	CC1
S071	45	10	14	8	6	10	3	2	CC45
S072	278	1	14	8	6	10	3	2	CC45
S073	45	10	14	8	6	10	3	2	CC45
S074	278	1	14	8	6	10	3	2	CC45
S075	610	1	1	1	1	4	1	1	CC1
S076	45	10	14	8	6	10	3	2	CC45
S077	278	1	14	8	6	10	3	2	CC45
S078	45	10	14	8	6	10	3	2	CC45
S079	199	13	13	1	1	12	1	13	CC15
S080	new_ST	12	4	1	15	12	1	2	
S081	2259	151	1	1	34	175	180	169	nan
S082	88	22	1	14	23	12	4	31	nan
S083	1984	13	13	1	1	12	3	2	CC15
S084	199	13	13	1	1	12	1	13	CC15
S085	6954	22	1	14	23	12	1	31	nan
S086	411	10	14	8	1	1	3	2	CC45
S087	2404	3	13	1	1	12	11	13	CC15
S088	1774	10	1	1	8	1	1	2	CC1
S089	848	10	1	1	1	12	1	1	CC1
S090	4680	10	14	8	6	19	3	2	CC45
S091	1984	13	13	1	1	12	3	2	CC15
S092	400	1	1	1	2	12	1	1	CC1
S093	2259	151	1	1	34	175	180	169	nan
S094	88	22	1	14	23	12	4	31	nan
S095	871	13	13	1	1	1	1	13	CC15
S096	14	1	13	1	1	12	11	13	CC15
S097	72	1	4	1	8	4	4	3	CC8
S098	45	10	14	8	6	10	3	2	CC45
S099	546	10	14	1	6	10	3	2	CC45
S100	45	10	14	8	6	10	3	2	CC45
S101	320	10	13	8	6	10	3	2	CC45
S102	45	10	14	8	6	10	3	2	CC45
S103	617	3	14	8	6	10	3	2	CC45
S104	45	10	14	8	6	10	3	2	CC45

S. no	ST	arcC	aroE	glpF	gmk	pta	tpi	yqiL	clonal complex
S105	320	10	13	8	6	10	3	2	CC45
S106	6775	3	1	1	8	1	11	1	CC1
S107	546	10	14	1	6	10	3	2	CC45
S108	45	10	14	8	6	10	3	2	CC45
S109	45	10	14	8	6	10	3	2	CC45
S110	546	10	14	1	6	10	3	2	CC45
S111	320	10	13	8	6	10	3	2	CC45
S112	411	10	14	8	1	1	3	2	CC45
S113	546	10	14	1	6	10	3	2	CC45
S114	15	13	13	1	1	12	11	13	CC15
S115	45	10	14	8	6	10	3	2	CC45
S116	321	10	14	8	6	10	5	2	CC45
S117	319	10	14	8	2	10	3	2	CC45
S118	45	10	14	8	6	10	3	2	CC45
S119	45	10	14	8	6	10	3	2	CC45
S120	45	10	14	8	6	10	3	2	CC45
S121	411	10	14	8	1	1	3	2	CC45
S122	8141	10	14	8	6	12	3	2	CC45
S123	2372	1	13	1	1	12	1	13	CC15
S124	45	10	14	8	6	10	3	2	CC45
S125	148	1	4	1	4	12	1	2	CC5
S126	320	10	13	8	6	10	3	2	CC45
S127	546	10	14	1	6	10	3	2	CC45
S128	2655	1	14	1	6	10	3	2	CC45
S129	188	3	1	1	8	1	1	1	CC1
S130	2655	1	14	1	6	10	3	2	CC45
S131	8141	10	14	8	6	12	3	2	CC45
S132	188	3	1	1	8	1	1	1	CC1
S133	320	10	13	8	6	10	3	2	CC45
S134	278	1	14	8	6	10	3	2	CC45
S135	188	3	1	1	8	1	1	1	CC1
S136	188	3	1	1	8	1	1	1	CC1
S137	45	10	14	8	6	10	3	2	CC45
S138	new_ST	10	14	8	6	1	3	2	
S139	320	10	13	8	6	10	3	2	CC45

S. no	ST	arcC	aroE	glpF	gmk	pta	tpi	yqiL	clonal complex
S140	546	10	14	1	6	10	3	2	CC45
S141	45	10	14	8	6	10	3	2	CC45
S142	617	3	14	8	6	10	3	2	CC45
S143	320	10	13	8	6	10	3	2	CC45
S144	2655	1	14	1	6	10	3	2	CC45
S145	1860	1	4	1	8	4	1	3	CC5
S146	45	10	14	8	6	10	3	2	CC45
S147	617	3	14	8	6	10	3	2	CC45
S148	296	3	1	1	8	1	1	2	CC1
S149	1054	1	1	8	1	1	1	1	CC1
S150	411	10	14	8	1	1	3	2	CC45
S151	8563	10	4	1	6	10	3	2	CC45
S152	45	10	14	8	6	10	3	2	CC45
S153	296	3	1	1	8	1	1	2	CC1
S154	411	10	14	8	1	1	3	2	CC45
S155	411	10	14	8	1	1	3	2	CC45
S156	1984	13	13	1	1	12	3	2	CC15
S157	296	3	1	1	8	1	1	2	CC1
S158	45	10	14	8	6	10	3	2	CC45
S159	617	3	14	8	6	10	3	2	CC45
S160	45	10	14	8	6	10	3	2	CC45
S161	296	3	1	1	8	1	1	2	CC1
S162	188	3	1	1	8	1	1	1	CC1
S163	319	10	14	8	2	10	3	2	CC45
S164	45	10	14	8	6	10	3	2	CC45
S165	617	3	14	8	6	10	3	2	CC45
S166	45	10	14	8	6	10	3	2	CC45
S167	45	10	14	8	6	10	3	2	CC45
S168	567	10	1	1	1	1	1	1	CC1
S169	188	3	1	1	8	1	1	1	CC1
S170	188	3	1	1	8	1	1	1	CC1
S171	7907	3	1	14	8	1	1	1	CC1
S172	6775	3	1	1	8	1	11	1	CC1
S173	546	10	14	1	6	10	3	2	CC45
S174	546	10	14	1	6	10	3	2	CC45

S. no	ST	arcC	aroE	glpF	gmk	pta	tpi	yqiL	clonal complex
S175	2791	3	1	1	8	12	1	1	CC1
S176	188	3	1	1	8	1	1	1	CC1
S177	617	3	14	8	6	10	3	2	CC45
S178	320	10	13	8	6	10	3	2	CC45
S179	719	3	3	1	1	4	4	1	CC8
S180	6775	3	1	1	8	1	11	1	CC1
S181	296	3	1	1	8	1	1	2	CC1
S182	617	3	14	8	6	10	3	2	CC45
S183	320	10	13	8	6	10	3	2	CC45
S184	320	10	13	8	6	10	3	2	CC45
S185	546	10	14	1	6	10	3	2	CC45
S186	188	3	1	1	8	1	1	1	CC1
S187	45	10	14	8	6	10	3	2	CC45
S188	8141	10	14	8	6	12	3	2	CC45
S189	8141	10	14	8	6	12	3	2	CC45
S190	792	10	14	8	6	10	3	3	CC45
S191	278	1	14	8	6	10	3	2	CC45
S192	794	10	2	8	6	10	3	2	CC45
S193	45	10	14	8	6	10	3	2	CC45
S194	45	10	14	8	6	10	3	2	CC45
S195	278	1	14	8	6	10	3	2	CC45
S196	45	10	14	8	6	10	3	2	CC45
S197	546	10	14	1	6	10	3	2	CC45
S198	546	10	14	1	6	10	3	2	CC45
S199	296	3	1	1	8	1	1	2	CC1
S200	546	10	14	1	6	10	3	2	CC45
S201	617	3	14	8	6	10	3	2	CC45
S202	45	10	14	8	6	10	3	2	CC45
S203	1774	10	1	1	8	1	1	2	CC1
S204	188	3	1	1	8	1	1	1	CC1
S205	8141	10	14	8	6	12	3	2	CC45
S206	14	1	13	1	1	12	11	13	CC15
S207	1724	3	13	1	8	1	1	1	CC1
S208	617	3	14	8	6	10	3	2	CC45
S209	792	10	14	8	6	10	3	3	CC45

S. no	ST	arcC	aroE	glpF	gmk	pta	tpi	yqiL	clonal complex
S210	296	3	1	1	8	1	1	2	CC1
S211	188	3	1	1	8	1	1	1	CC1
S212	1505	1	1	1	8	12	1	1	CC1
S213	296	3	1	1	8	1	1	2	CC1
S214	260	1	1	1	1	12	11	13	CC15
S215	1724	3	13	1	8	1	1	1	CC1
S216	400	1	1	1	2	12	1	1	CC1
S217	296	3	1	1	8	1	1	2	CC1
S218	567	10	1	1	1	1	1	1	CC1
S219	296	3	1	1	8	1	1	2	CC1
S220	610	1	1	1	1	4	1	1	CC1
S221	1906	13	13	1	1	10	11	13	CC15
S222	546	10	14	1	6	10	3	2	CC45

Table S5: Multidrug Resistance Patterns of MRSA and MSSA isolates across different ST

spid	MRSA	Resistant antibiotics	Num resistant	ST	Clonal complex
S03-02-04	MRSA	GEN, CIP, LVX, MFX, ERY, CLI, TCY, SXT, AMP	9	6775	CC1
S04-03-06	MRSA	GEN, CIP, LVX, MFX, ERY, CLI, TCY, SXT, AMP	9	6775	CC1
S01-11-02	MRSA	GEN, CIP, LVX, MFX, ERY, CLI, TCY, SXT	8	2139	CC1
S03-01-10	MSSA	GEN, CIP, LVX, MFX, ERY, CLI, SXT, AMP	8	617	CC45
S03-06-12	MRSA	GEN, CIP, LVX, MFX, ERY, CLI, TCY, SXT	8	188	CC1
S03-07-05	MSSA	GEN, CIP, LVX, MFX, ERY, CLI, SXT, AMP	8	188	CC1
S04-01-22	MRSA	GEN, CIP, LVX, MFX, ERY, CLI, SXT, AMP	8	296	CC1
S04-02-20	MSSA	GEN, CIP, LVX, MFX, ERY, CLI, SXT, AMP	8	567	CC1
S04-02-25	MSSA	GEN, CIP, LVX, MFX, ERY, CLI, SXT, AMP	8	188	CC1
S04-03-18	MSSA	GEN, CIP, LVX, MFX, ERY, CLI, SXT, AMP	8	188	CC1
S04-05-11	MSSA	GEN, CIP, LVX, MFX, ERY, CLI, SXT, AMP	8	188	CC1
S02-01-07	MRSA	GEN, CIP, LVX, MFX, ERY, CLI, SXT	7	188	CC1
S02-01-08	MSSA	GEN, CIP, LVX, MFX, ERY, CLI, SXT	7	188	CC1
S02-03-23	MSSA	GEN, CIP, LVX, MFX, ERY, CLI, SXT	7	188	CC1
S02-04-09	MRSA	GEN, CIP, LVX, MFX, ERY, CLI, SXT	7	188	CC1
S02-04-11	MRSA	GEN, CIP, LVX, MFX, ERY, CLI, SXT	7	188	CC1
S02-07-06	MSSA	GEN, CIP, LVX, MFX, ERY, CLI, SXT	7	new_ST	
S02-09-08	MSSA	GEN, CIP, LVX, MFX, ERY, CLI, SXT	7	411	CC45
S03-04-18	MSSA	GEN, CIP, LVX, MFX, ERY, CLI, SXT	7	148	CC5
S03-05-17	MRSA	GEN, CIP, LVX, MFX, ERY, CLI, SXT	7	188	CC1
S03-08-06	MSSA	GEN, CIP, LVX, ERY, CLI, TCY, SXT	7	546	CC45
S03-09-07	MSSA	CIP, LVX, MFX, ERY, CLI, TCY, SXT	7	2655	CC45
S03-09-25	MRSA	GEN, CIP, LVX, MFX, ERY, CLI, SXT	7	617	CC45

spid	MRSA	Resistant antibiotics	Num resistant	ST	Clonal complex
S03-10-14	MRSA	GEN, CIP, LVX, MFX, ERY, CLI, SXT	7	296	CC1
S03-11-08	MSSA	GEN, CIP, LVX, ERY, CLI, SXT, AMP	7	1054	CC1
S04-01-05	MSSA	GEN, CIP, LVX, MFX, ERY, CLI, SXT	7	296	CC1
S04-02-03	MRSA	GEN, CIP, LVX, MFX, ERY, CLI, SXT	7	188	CC1
S04-03-02	MSSA	GEN, CIP, LVX, MFX, ERY, CLI, SXT	7	188	CC1
S04-04-21	MSSA	GEN, CIP, LVX, MFX, ERY, CLI, SXT	7	296	CC1
S04-08-23	MRSA	GEN, CIP, LVX, MFX, ERY, CLI, SXT	7	188	CC1
S04-10-08	MSSA	GEN, CIP, LVX, MFX, ERY, CLI, SXT	7	1505	CC1
S04-11-09	MRSA	GEN, CIP, LVX, MFX, ERY, CLI, SXT	7	296	CC1
S01-08-09	MRSA	GEN, CIP, LVX, ERY, CLI, SXT	6	2201	CC1
S03-07-09	MSSA	GEN, ERY, CLI, TCY, SXT, AMP	6	188	CC1
S03-09-05	MSSA	GEN, CIP, LVX, ERY, TCY, SXT	6	320	CC45
S01-06-07	MRSA	GEN, ERY, CLI, TCY, SXT	5	264	CC1
S02-02-13	MSSA	GEN, ERY, CLI, TCY, SXT	5	411	CC45
S02-03-18	MSSA	GEN, ERY, CLI, TCY, SXT	5	2261	nan
S02-04-25	MSSA	GEN, ERY, CLI, TCY, SXT	5	2261	nan
S02-11-12	MSSA	GEN, ERY, CLI, TCY, SXT	5	88	nan
S02-12-18	MSSA	GEN, ERY, CLI, SXT, DAP	5	45	CC45
S03-01-06	MRSA	GEN, ERY, CLI, SXT, AMP	5	320	CC45
S03-02-01	MRSA	GEN, ERY, CLI, SXT, AMP	5	320	CC45
S03-03-13	MRSA	GEN, ERY, CLI, TCY, SXT	5	319	CC45
S03-04-07	MSSA	GEN, ERY, CLI, SXT, AMP	5	411	CC45
S03-04-10	MSSA	GEN, CIP, LVX, MFX, SXT	5	8141	CC45
S04-10-20	MRSA	ERY, CLI, TCY, SXT, AMP	5	400	CC1
S04-11-19	MSSA	ERY, CLI, TCY, SXT, AMP	5	567	CC1
S01-04-16	MSSA	GEN, ERY, CLI, SXT	4	1774	CC1
S01-06-09	MSSA	GEN, ERY, CLI, SXT	4	278	CC45
S01-11-23	MSSA	GEN, ERY, CLI, SXT	4	320	CC45
S01-12-07	MSSA	GEN, ERY, CLI, SXT	4	new_ST	
S02-01-10	MSSA	GEN, ERY, CLI, SXT	4	2655	CC45
S02-01-16	MSSA	GEN, ERY, CLI, SXT	4	14	CC15
S02-02-18	MSSA	GEN, ERY, TCY, SXT	4	1659	CC15

spid	MRSA	Resistant antibiotics	Num resistant	ST	Clonal complex
S02-03-04	MSSA	GEN, ERY, CLI, SXT	4	2259	nan
S02-03-12	MSSA	GEN, ERY, TCY, SXT	4	15	CC15
S02-03-19	MSSA	GEN, ERY, CLI, SXT	4	2261	nan
S02-03-20	MSSA	GEN, ERY, CLI, SXT	4	2259	nan
S02-03-25	MSSA	GEN, ERY, CLI, SXT	4	2261	nan
S02-04-15	MRSA	ERY, CLI, TCY, SXT	4	1232	nan
S02-05-03	MSSA	GEN, ERY, CLI, SXT	4	1984	CC15
S02-05-05	MRSA	ERY, CLI, TCY, SXT	4	617	CC45
S02-05-06	MRSA	ERY, CLI, TCY, SXT	4	1232	nan
S02-05-17	MSSA	GEN, ERY, CLI, SXT	4	1	CC1
S02-08-01	MSSA	ERY, CLI, TCY, SXT	4	199	CC15
S02-09-10	MRSA	ERY, CLI, TCY, SXT	4	2404	CC15
S02-11-07	MSSA	GEN, ERY, CLI, SXT	4	2259	nan
S02-12-06	MSSA	GEN, ERY, TCY, SXT	4	14	CC15
S03-01-09	MRSA	ERY, CLI, SXT, AMP	4	45	CC45
S03-02-17	MSSA	GEN, ERY, CLI, SXT	4	546	CC45
S03-03-03	MSSA	GEN, ERY, TCY, SXT	4	15	CC15
S03-04-11	MSSA	GEN, CIP, LVX, SXT	4	2372	CC15
S03-06-21	MSSA	GEN, ERY, TCY, SXT	4	278	CC45
S03-08-04	MSSA	GEN, ERY, CLI, SXT	4	320	CC45
S03-09-04	MSSA	GEN, ERY, CLI, SXT	4	617	CC45
S03-11-24	MRSA	GEN, ERY, CLI, SXT	4	8563	CC45
S03-12-06	MSSA	GEN, ERY, CLI, SXT	4	296	CC1
S03-12-14	MRSA	GEN, ERY, CLI, SXT	4	411	CC45
S04-01-12	MSSA	GEN, ERY, CLI, SXT	4	45	CC45
S04-02-15	MSSA	ERY, CLI, TCY, SXT	4	617	CC45
S04-03-19	MSSA	GEN, ERY, CLI, SXT	4	617	CC45
S04-04-04	MSSA	GEN, ERY, TCY, SXT	4	320	CC45
S04-05-13	MSSA	ERY, CLI, TCY, SXT	4	45	CC45
S04-08-12	MSSA	GEN, ERY, CLI, SXT	4	1774	CC1
S04-09-09	MSSA	GEN, ERY, TCY, SXT	4	14	CC15
S04-10-16	MSSA	GEN, ERY, TCY, SXT	4	1724	CC1
S04-12-06	MSSA	GEN, ERY, CLI, SXT	4	296	CC1
S04-12-17	MSSA	ERY, CLI, TCY, SXT	4	610	CC1
S02-01-01	MRSA	ERY, CLI, SXT	3	45	CC45
S02-01-23	MRSA	ERY, CLI, SXT	3	546	CC45
S02-02-08	MRSA	ERY, CLI, SXT	3	45	CC45
S02-03-10	MSSA	ERY, CLI, SXT	3	617	CC45

spid	MRSA	Resistant antibiotics	Num resistant	ST	Clonal complex
S02-03-21	MSSA	GEN, TCY, SXT	3	7	nan
S02-04-02	MRSA	ERY, CLI, SXT	3	45	CC45
S02-04-21	MSSA	CIP, LVX, SXT	3	188	CC1
S02-05-21	MRSA	ERY, CLI, SXT	3	278	CC45
S02-08-24	MSSA	GEN, TCY, SXT	3	6954	nan
S02-09-13	MSSA	ERY, CLI, SXT	3	1774	CC1
S03-01-18	MSSA	ERY, CLI, SXT	3	45	CC45
S03-02-08	MSSA	ERY, SXT, AMP	3	45	CC45
S03-05-08	MRSA	ERY, CLI, SXT	3	2655	CC45
S03-07-24	MSSA	GEN, TCY, SXT	3	new_ST	
S04-01-08	MRSA	ERY, CLI, SXT	3	45	CC45
S04-02-17	MSSA	ERY, CLI, SXT	3	45	CC45
S04-03-08	MSSA	ERY, CLI, SXT	3	546	CC45
S04-05-15	MRSA	ERY, CLI, SXT	3	8141	CC45
S04-05-18	MSSA	ERY, CLI, SXT	3	278	CC45
S04-06-18	MRSA	ERY, CLI, SXT	3	546	CC45
S04-08-06	MSSA	ERY, CLI, SXT	3	45	CC45
S04-12-23	MSSA	ERY, TCY, SXT	3	1906	CC15
S01-01-10	MSSA	ERY, SXT	2	319	CC45
S01-01-21	MRSA	ERY, SXT	2	45	CC45
S01-01-22	MRSA	ERY, SXT	2	45	CC45
S01-02-24	MRSA	ERY, SXT	2	45	CC45
S01-04-01	MRSA	ERY, SXT	2	45	CC45
S02-01-06	MRSA	ERY, SXT	2	546	CC45
S02-01-12	MRSA	ERY, SXT	2	546	CC45
S02-01-18	MRSA	ERY, SXT	2	546	CC45
S02-01-22	MRSA	ERY, SXT	2	1774	CC1
S02-02-09	MRSA	ERY, SXT	2	546	CC45
S02-02-15	MRSA	ERY, SXT	2	45	CC45
S02-02-17	MRSA	ERY, SXT	2	546	CC45
S02-03-07	MRSA	ERY, SXT	2	546	CC45
S02-03-24	MSSA	ERY, SXT	2	45	CC45
S02-04-06	MSSA	GEN, SXT	2	805	CC1
S02-05-22	MRSA	ERY, SXT	2	45	CC45
S02-06-13	MSSA	ERY, SXT	2	45	CC45
S02-07-12	MSSA	TCY, SXT	2	88	nan
S02-10-18	MSSA	ERY, SXT	2	848	CC1
S02-10-20	MRSA	ERY, SXT	2	4680	CC45

spid	MRSA	Resistant antibiotics	Num resistant	ST	Clonal complex
S02-10-22	MSSA	ERY, SXT	2	1984	CC15
S02-10-26	MRSA	TCY, SXT	2	400	CC1
S02-12-14	MSSA	GEN, SXT	2	72	CC8
S03-01-02	MRSA	ERY, SXT	2	546	CC45
S03-01-03	MRSA	ERY, SXT	2	45	CC45
S03-02-06	MRSA	ERY, SXT	2	546	CC45
S03-02-07	MRSA	ERY, SXT	2	45	CC45
S03-02-19	MRSA	ERY, SXT	2	320	CC45
S03-03-09	MRSA	ERY, SXT	2	45	CC45
S03-04-15	MSSA	ERY, SXT	2	45	CC45
S03-06-14	MRSA	ERY, SXT	2	320	CC45
S03-09-08	MSSA	GEN, SXT	2	1860	CC5
S04-01-01	MRSA	ERY, SXT	2	411	CC45
S04-01-04	MRSA	ERY, SXT	2	1984	CC15
S04-01-09	MRSA	ERY, SXT	2	617	CC45
S04-03-11	MRSA	ERY, SXT	2	546	CC45
S04-05-03	MSSA	ERY, SXT	2	320	CC45
S04-05-04	MSSA	ERY, SXT	2	320	CC45
S04-05-05	MSSA	ERY, SXT	2	546	CC45
S04-05-23	MRSA	ERY, SXT	2	45	CC45
S04-06-01	MRSA	SXT, DAP	2	45	CC45
S04-06-04	MSSA	ERY, SXT	2	278	CC45
S04-07-14	MSSA	SXT, DAP	2	546	CC45
S04-07-16	MSSA	TCY, SXT	2	617	CC45
S04-09-10	MSSA	ERY, SXT	2	1724	CC1
S04-09-22	MSSA	ERY, SXT	2	792	CC45
S04-10-09	MSSA	GEN, SXT	2	296	CC1
S01-01-14	MRSA	SXT	1	546	CC45
S01-01-17	MSSA	SXT	1	121	CC121
S01-04-05	MRSA	SXT	1	546	CC45
S01-04-09	MSSA	SXT	1	new_ST	
S01-05-14	MRSA	SXT	1	546	CC45
S01-06-11	MSSA	SXT	1	319	CC45
S01-06-22	MRSA	SXT	1	546	CC45
S01-08-01	MSSA	SXT	1	320	CC45
S01-08-17	MRSA	SXT	1	45	CC45
S01-09-23	MRSA	SXT	1	320	CC45
S01-12-05	MSSA	SXT	1	2125	CC1

spid	MRSA	Resistant antibiotics	Num resistant	ST	Clonal complex
S02-01-09	MSSA	SXT	1	319	CC45
S02-01-13	MRSA	SXT	1	320	CC45
S02-02-04	MSSA	SXT	1	45	CC45
S02-02-19	MRSA	SXT	1	45	CC45
S02-02-24	MRSA	SXT	1	2231	CC45
S02-03-01	MSSA	SXT	1	97	CC97
S02-04-01	MRSA	SXT	1	278	CC45
S02-05-15	MSSA	SXT	1	2404	CC15
S02-05-20	MRSA	SXT	1	45	CC45
S02-05-24	MRSA	SXT	1	278	CC45
S02-06-02	MSSA	SXT	1	610	CC1
S02-06-07	MRSA	SXT	1	45	CC45
S02-06-10	MRSA	SXT	1	278	CC45
S02-06-21	MSSA	SXT	1	199	CC15
S02-07-07	MSSA	SXT	1	2259	nan
S02-07-23	MSSA	SXT	1	1984	CC15
S02-11-14	MSSA	SXT	1	871	CC15
S03-02-23	MSSA	SXT	1	411	CC45
S03-02-25	MRSA	SXT	1	546	CC45
S03-03-10	MRSA	SXT	1	321	CC45
S03-03-19	MSSA	SXT	1	45	CC45
S03-03-22	MRSA	SXT	1	45	CC45
S03-04-06	MRSA	SXT	1	45	CC45
S03-05-04	MRSA	SXT	1	320	CC45
S03-05-05	MRSA	SXT	1	546	CC45
S03-06-01	MSSA	SXT	1	2655	CC45
S03-06-08	MRSA	SXT	1	8141	CC45
S03-07-17	MSSA	SXT	1	45	CC45
S03-08-13	MRSA	SXT	1	45	CC45
S03-09-18	MSSA	SXT	1	45	CC45
S03-11-15	MRSA	SXT	1	411	CC45
S03-12-05	MRSA	SXT	1	45	CC45
S04-02-06	MSSA	SXT	1	319	CC45
S04-02-09	MSSA	SXT	1	45	CC45
S04-02-18	MSSA	SXT	1	45	CC45
S04-03-04	MSSA	SXT	1	7907	CC1
S04-03-17	MSSA	SXT	1	2791	CC1
S04-04-15	MSSA	SXT	1	719	CC8

spid	MRSA	Resistant antibiotics	Num resistant	ST	Clonal complex
S04-04-17	MSSA	SXT	1	6775	CC1
S04-02-08	MSSA	SXT	1	617	CC45
S04-05-14	MSSA	SXT	1	8141	CC45
S04-05-16	MSSA	SXT	1	792	CC45
S04-05-19	MSSA	SXT	1	794	CC45
S04-06-06	MRSA	SXT	1	45	CC45
S04-06-16	MRSA	SXT	1	546	CC45
S04-07-06	MSSA	SXT	1	296	CC1
S04-09-01	MSSA	SXT	1	8141	CC45
S04-09-16	MSSA	SXT	1	617	CC45
S04-10-05	MSSA	SXT	1	296	CC1
S04-10-06	MSSA	SXT	1	188	CC1
S04-10-14	MSSA	SXT	1	260	CC15

Figure S3: Visualize associated isolate data on the eBURST image

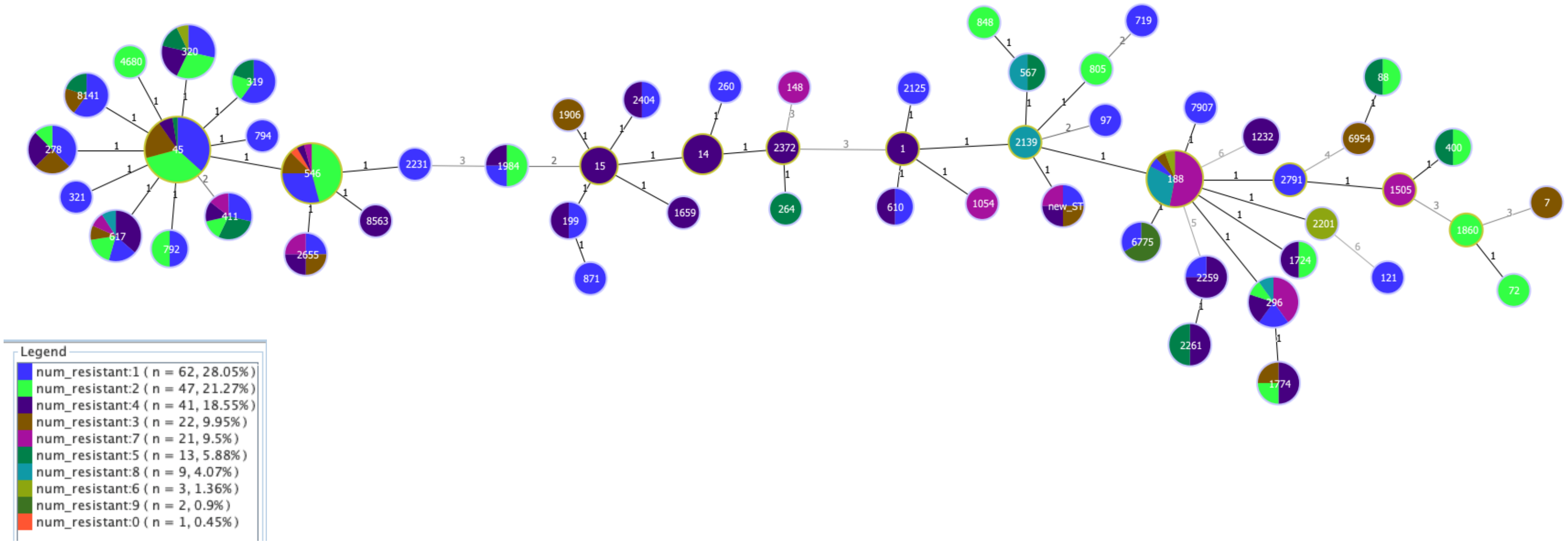

Supplement: Supplementary file 1 [file pathogens-15-00238-s001.zip › pathogens-4102753-supplementary.pdf]
